# The Restrictive Red Blood Cell Transfusion Strategy for Critically Injured Patients (RESTRIC) trial: a cluster-randomized, crossover, non-inferiority multicenter trial of restrictive transfusion in trauma

**DOI:** 10.1186/s40560-023-00682-3

**Published:** 2023-07-24

**Authors:** Mineji Hayakawa, Takashi Tagami, Daisuke Kudo, Kota Ono, Makoto Aoki, Akira Endo, Tetsuya Yumoto, Yosuke Matsumura, Shiho Irino, Kazuhiko Sekine, Noritaka Ushio, Takayuki Ogura, Sho Nachi, Yuhei Irie, Katsura Hayakawa, Yusuke Ito, Yuko Okishio, Tomohiro Muronoi, Yoshinori Kosaki, Kaori Ito, Keita Nakatsutsumi, Yutaka Kondo, Taichiro Ueda, Hiroshi Fukuma, Yuichi Saisaka, Naoki Tominaga, Takeo Kurita, Fumihiko Nakayama, Tomotaka Shibata, Shigeki Kushimoto

**Affiliations:** 1 Department of Emergency Medicine, Hokkaido University Hospital, N14W5 Kita-ku, Sapporo, 060-8648 Japan; 2 Department of Emergency and Critical Care Medicine, Nippon Medical School Musashi Kosugi Hospital, Kawasaki, Japan; 3 Department of Clinical Epidemiology and Health Economics, School of Public Health, The University of Tokyo, Tokyo, Japan; 4 Division of Emergency and Critical Care Medicine, Tohoku University Graduate School of Medicine, Sendai, Japan; 5 Ono Biostat Consulting, Tokyo, Japan; 6 Department of Emergency Medicine, Gunma University Graduate School of Medicine, Maebashi, Japan; 7 Department of Acute Critical Care and Disaster Medicine, Tokyo Medical and Dental University Graduate School of Medicine, Tokyo, Japan; 8 Department of Emergency, Critical Care, and Disaster Medicine, Okayama University Graduate School of Medicine, Dentistry and Pharmaceutical Sciences, Okayama, Japan; 9 Department of Intensive Care, Chiba Emergency Medical Centre, Chiba, Japan; 10 Department of Emergency and Critical Care Medicine, Tokyo Saiseikai Central Hospital, Tokyo, Japan; 11 Department of Emergency and Critical Care Medicine, Japan Red Cross Maebashi Hospital, Maebashi, Japan; 12 Department of Emergency Medicine and Critical Care Medicine, Tochigi Prefectural Emergency and Critical Care Centre, Imperial Gift Foundation Saiseikai, Utsunomiya Hospital, Utsunomiya, Japan; 13 Advanced Critical Care Centre, Gifu University Hospital, Gifu, Japan; 14 Department of Emergency and Critical Care Medicine, Fukuoka University Hospital, Fukuoka, Japan; 15 Advanced Emergency and Critical Care Centre, Saitama Red Cross Hospital, Saitama, Japan; 16 Senri Critical Care Medical Centre, Saiseikai Senri Hospital, Suita, Japan; 17 Department of Emergency and Critical Care Medicine, Wakayama Medical University, Wakayama, Japan; 18 Department of Acute Care Surgery, Shimane University Faculty of Medicine, Izumo, Japan; 19 Department of Surgery, Division of Acute Care Surgery, Teikyo University School of Medicine, Tokyo, Japan; 20 Trauma and Acute Critical Care Centre, Tokyo Medical and Dental University Hospital, Tokyo, Japan; 21 Department of Emergency and Critical Care Medicine, Juntendo University Urayasu Hospital, Urayasu, Japan; 22 Shock and Trauma Centre, Nippon Medical School Chiba Hokusoh Hospital, Inzai, Japan; 23 Senshu Trauma and Critical Care Centre, Rinku General Medical Centre, Izumisano, Japan; 24 Emergency and Critical Care Centre, Kochi Health Sciences Centre, Kochi, Japan; 25 Department of Emergency and Critical Care Medicine, Nippon Medical School, Tokyo, Japan; 26 Department of Emergency and Critical Care Medicine, Chiba University Graduate School of Medicine, Chiba, Japan; 27 Department of Emergency and Critical Care Medicine, Nippon Medical School Tama Nagayama Hospital, Tama, Japan; 28 Advanced Trauma, Emergency and Critical Care Centre, Oita University Hospital, Yufu, Japan

**Keywords:** Resuscitation, Red blood cell, Hemoglobin, Trauma, Transfusion

## Abstract

**Background:**

The efficacies of fresh frozen plasma and coagulation factor transfusion have been widely evaluated in trauma-induced coagulopathy management during the acute post-injury phase. However, the efficacy of red blood cell transfusion has not been adequately investigated in patients with severe trauma, and the optimal hemoglobin target level during the acute post-injury and resuscitation phases remains unclear. Therefore, this study aimed to examine whether a restrictive transfusion strategy was clinically non-inferior to a liberal transfusion strategy during the acute post-injury phase.

**Methods:**

This cluster-randomized, crossover, non-inferiority multicenter trial was conducted at 22 tertiary emergency medical institutions in Japan and included adult patients with severe trauma at risk of major bleeding. The institutions were allocated a restrictive or liberal transfusion strategy (target hemoglobin levels: 7–9 or 10–12 g/dL, respectively). The strategies were applied to patients immediately after arrival at the emergency department. The primary outcome was 28-day survival after arrival at the emergency department. Secondary outcomes included transfusion volume, complication rates, and event-free days. The non-inferiority margin was set at 3%.

**Results:**

The 28-day survival rates of patients in the restrictive (*n* = 216) and liberal (*n* = 195) strategy groups were 92.1% and 91.3%, respectively. The adjusted odds ratio for 28-day survival in the restrictive versus liberal strategy group was 1.02 (95% confidence interval: 0.49–2.13). Significant non-inferiority was not observed. Transfusion volumes and hemoglobin levels were lower in the restrictive strategy group than in the liberal strategy group. No between-group differences were noted in complication rates or event-free days.

**Conclusions:**

Although non-inferiority of the restrictive versus liberal transfusion strategy for 28-day survival was not statistically significant, the mortality and complication rates were similar between the groups. The restrictive transfusion strategy results in a lower transfusion volume.

*Trial registration number:* umin.ac.jp/ctr: UMIN000034405, registration date: 8 October 2018.

**Supplementary Information:**

The online version contains supplementary material available at 10.1186/s40560-023-00682-3.

## Background

The efficacies of fresh frozen plasma (FFP) and coagulation factor transfusion have been widely evaluated in trauma-induced coagulopathy management during the acute post-injury phase [1, 2]. However, the efficacy of red blood cell (RBC) transfusion has not been adequately investigated in patients with severe trauma, and the optimal hemoglobin target level during the acute post-injury and resuscitation phases remains unclear.

The fifth edition of the European guidelines on the management of major bleeding and coagulopathy following trauma, the most recent international guidelines, recommends target hemoglobin levels of 7–9 g/dL [2]. This recommendation is based on the post-hoc analysis results of the Transfusion Requirements in Critical Care (TRICC) trial [3], which compared the efficacies of a restrictive versus liberal RBC transfusion strategy (target hemoglobin levels: 7–9 or 10–12 g/dL, respectively) in patients admitted to the intensive care unit (ICU) [4]. However, the study interventions were applied after ICU admission [4]. Furthermore, patients with active bleeding were excluded [4]. Therefore, it is inappropriate to apply these trial and post-hoc analysis findings to patients in the acute phase of severe trauma [3, 4]. Moreover, the rationale section of the European guidelines [2] emphasizes that the TRICC trial [4] was neither designed nor powered to precisely determine the target hemoglobin level.

In this Restrictive Transfusion Strategy for Critically Injured Patients (RESTRIC) trial, we examined whether a restrictive RBC transfusion strategy was clinically non-inferior to a liberal RBC transfusion strategy in patients with severe trauma at risk of active bleeding during the acute post-injury phase.

## Methods

### Design and setting

The RESTRIC trial was a cluster-randomized, crossover, non-inferiority multicenter trial of patients with severe trauma and was registered with the UMIN Clinical Trials Registry (UMIN000034405) on October 8, 2018. The protocol (V.1.3) was initially approved on October 11, 2018. The detailed trial protocol was published in July 2020 [5]. The original protocol is in Japanese but was translated into English (Additional file 1). This pragmatic trial aimed to reproduce real-world management of how a transfusion strategy is applied upon patient arrival at the ED based as far as possible on the physician’s judgment. In this trial, we applied a cluster-randomized design to enable the study intervention initiation upon patient arrival at the ED; furthermore, the crossover design was implemented to reduce the confounding effects of different hospitals. The study protocol and statistical analysis plan were previously published [5]. All procedures performed in studies involving human participants were in accordance with the ethical standards of the institutional and/or national research committee and with the 1964 Declaration of Helsinki and its later amendments or comparable ethical standards. The study design was approved by the Ethics Committee of each participating institution (Additional file 2) and that of the Japanese Association for the Surgery of Trauma.

**Fig. 1:**
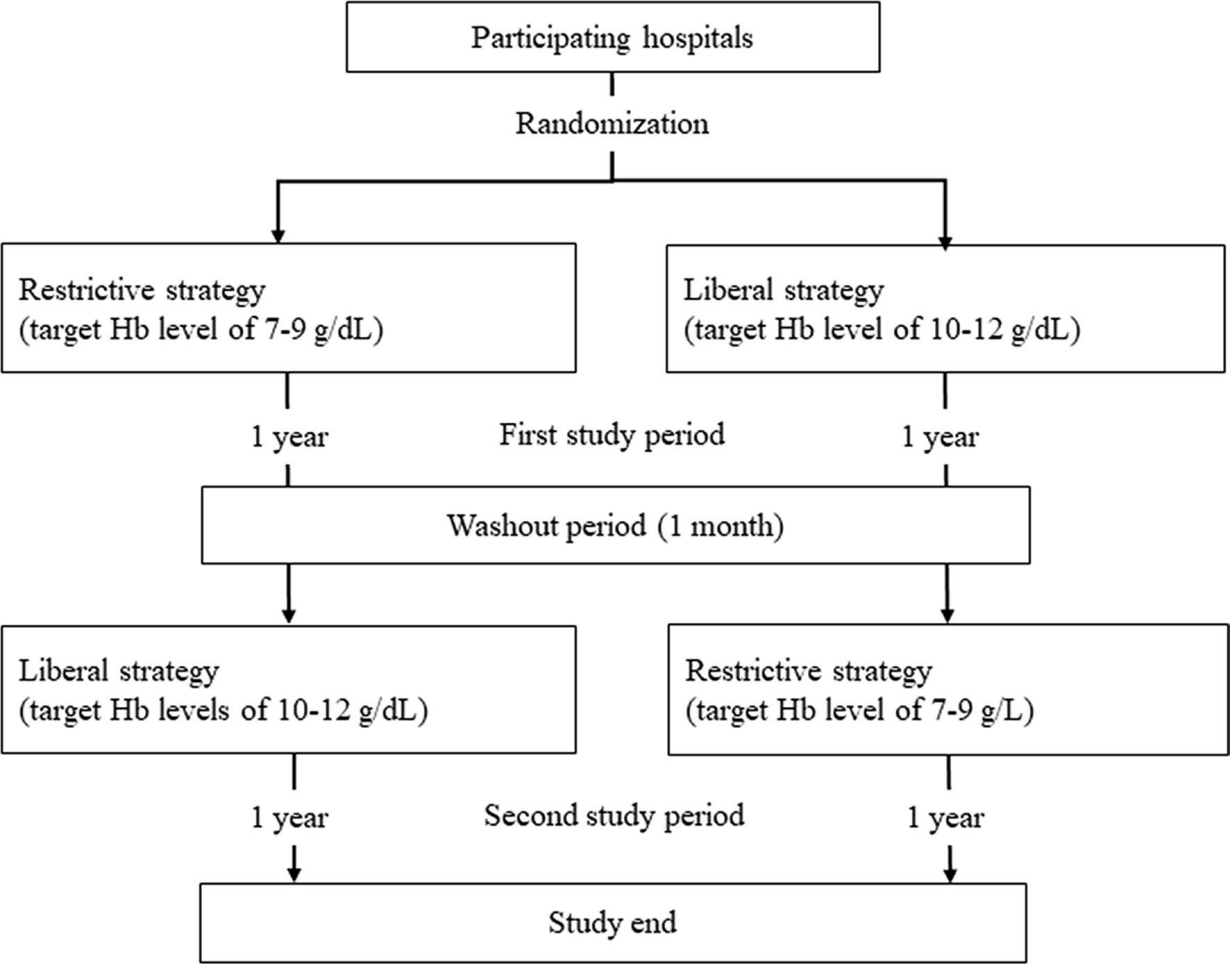
Study design. A total of 22 emergency medical centers participated in the RESTRIC trial. The participating institutions were randomized to implement either the restrictive or liberal RBC transfusion strategy at a ratio of 1:1. *Hb* hemoglobin, *RBC* red blood cell, *RESTRIC* Restrictive Transfusion Strategy for Critically Injured Patients

Japanese tertiary emergency medical centers participated in the RESTRIC trial. The participating institutions were randomized to two schedules (restrictive or liberal RBC transfusion strategy [target hemoglobin levels: 7–9 or 10–12 g/dL, respectively]) with a 1:1 ratio based on a pre-created random assignment table. After randomization, the centers applied the first transfusion strategy for 1 year (first study period). After a 1-month washout period following the first study period, the second transfusion strategy was applied for another year (second study period) (Fig. 1).

The allocated transfusion strategy was posted at each center to provide opt-out opportunities to patients and their next-of-kin. The allocated transfusion strategy was applied to all patients during the initial trauma resuscitation phase and upon arrival at the ED. Written informed consent was obtained as soon as possible from the patients or their next-of-kin, after which the patients were enrolled in the trial; thereafter, the transfusion strategy was applied until a predefined initiation and follow-up period. The applied transfusion strategy was selected at the physician’s discretion for patients who declined enrolment in the trial.

### Participants

The need for RBC transfusion is not always apparent at the time of arrival at the ED. Therefore, we included patients with trauma, aged ≥ 20 years, who had one of the following complications or conditions, based on the physician’s judgment: severe bleeding that can result in circulatory shock; suspected severe bleeding after arrival at the ED; and the potential for severe bleeding postoperatively during the acute phase of trauma. Furthermore, we excluded patients based on the following criteria: cardiac arrest before or upon arrival at the ED; transfer from another hospital; the physician’s decision to withdraw active treatment at initial assessment; complications of severe burns (≥ 15% body surface area); pregnancy; chronic anemia, as determined by the attending physician based on medical history (hemoglobin level: ≤ 7 g/dL); and objection to blood transfusion.

### Intervention and follow-up

RBC transfusion is often initiated in patients with severe trauma with active bleeding before confirming a decrease in hemoglobin levels. Therefore, each RBC transfusion strategy was defined based on the target hemoglobin level rather than the threshold hemoglobin level. The attending physician determined the RBC transfusion initiation timing in patients with active bleeding based on hemoglobin levels and the presence of hemodynamic instability. Notably, the restrictive RBC transfusion strategy was not permissive hypotension and hypovolemia resuscitation strategy. When the hemoglobin level was sufficiently high as the target of each strategy, a crystalloid and/or colloid were administered for resuscitation. One RBC transfusion strategy was applied until 7 days after hospital admission, discharge from the ICU, decision to withdraw active treatment, or death. Patients were followed up for 28 days. Investigators contacted patients (or their representatives, as appropriate) discharged from the hospital prior to 28 days after arrival at the ED by telephone to collect information regarding patient status.

### Safety monitoring

A safety monitoring board, comprising two independent experts not involved in the trial, was responsible for monitoring trial safety. Significant adverse events were immediately recorded in the medical record and electronic data capture system, the same system that recorded the assessment data. The treating physician reported significant adverse events to the site investigator, who reported them to the chief investigator of each site and the principal investigator. The principal investigator consulted the safety monitoring board. The board reviewed and examined the report and sent written recommendations in response to the principal investigator.

### Outcomes

To evaluate the non-inferiority of the restrictive RBC transfusion strategy to the liberal RBC transfusion strategy, 28-day survival after arrival at the ED was used as the primary outcome. Patients with incomplete information regarding survival or death 28 days after arrival at the ED were considered dropouts and excluded from the analysis. Secondary outcomes included the following: time to death during the first 28 days; cumulative RBC concentrate, FFP, and platelet concentrate volumes transfused on Days 1, 7, and 28; ventilator-, catecholamine-, and ICU-free days during the first 28 days; organ (renal, hepatic, and respiratory) failure during the first 7 days; complication (deep venous thrombosis, pulmonary embolism, cerebral infarction, myocardial infarction, bowel ischemia, transfusion-associated lung injury [6], and sepsis [7]) rates during the first 28 days; and Glasgow Outcome Scale scores at hospital discharge. The number of event-free days for patients who died during the first 28 days after arrival at the ED was zero. Renal failure was defined as Stage III, according to the Kidney Disease: Improving Global Outcomes guidelines [8]. Hepatic failure was defined as a total bilirubin level ≥ 6 mg/dL, based on the Sequential Organ Failure Assessment score [9]. Respiratory failure was defined as moderate acute respiratory distress syndrome, according to the Berlin definition [10].

### Sample size

Based on our previous retrospective multicenter observational study [11–18], we assumed a 25% mortality rate at 28 days after arrival at the ED in patients exposed to the liberal RBC transfusion strategy. The inter-class and inter-period correlation coefficients were set at 0.05, and the non-inferiority margin was set at 3%. The non-inferiority margin was defined based on statistically and clinically acceptable tolerance margins, as referenced in previous large-scale clinical trials [4, 19–21]. In the RESTRIC trial protocol, we calculated a sample size of 170 patients for each of the transfusion (restrictive and liberal RBC) strategy groups to reach a power of 80% and a one-sided alpha of 2.5%, assuming that 17 centers participated as a cluster [5, 22]. Therefore, we set the target sample size at 400 patients, considering possible cluster size variation, including non-eligible patients and dropouts during follow-up [5]. However, the actual sample size required (6214 patients) was much larger than the target sample size (400 patients) because of an error in the sample size calculation [22]. Because the error was not discovered until the end of the study, the study could not be terminated during its course.

### Statistical analyses

Continuous variables are expressed as medians (interquartile ranges) and were compared using the Wilcoxon rank sum test. Categorical variables are expressed as numbers and percentages and were compared using the χ^2^ test or Fisher’s exact test if the expected count was < 5. The primary outcome analysis was adjusted for clustering within sites. The analysis used a mixed model, with intervention (restrictive or liberal RBC transfusion strategy) and period (order of transfusion strategy allocation) as fixed effect factors. Site and interaction between site (participating institution) and period (order of transfusion strategy allocation) were incorporated as random effect factors [23]. Furthermore, the non-inferiority margin was set at 3%. The null hypothesis was P1–P0 ≤ –0.03 (P0, 28-day survival rate [liberal RBC transfusion strategy]; P1, 28-day survival rate [restrictive RBC transfusion strategy]). Therefore, we evaluated whether the lower limit of the P1–P0 95% confidence interval (CI) exceeded –0.03. However, a logistic regression model was used for the primary analysis. Thus, we converted the non-inferiority margin into a certain value in terms of the odds ratio, which was determined based on the actual value of P0. We evaluated whether the lower limit of the 95% CI of the odds ratio exceeded this value (Results section). After excluding cases with missing primary outcome data, we used the full analysis set for the primary outcome analysis. In particular, we followed the intention-to-treat and per-protocol analysis principles for the primary and sensitivity analyses, respectively. The per-protocol analysis excluded cases in which transfusions intentionally deviated from the target hemoglobin level. Subgroup analysis was performed to investigate the effect of the intervention in terms of sex, age (< 60 *vs*. ≥ 60 years), Injury Severity Score (< 16 *vs*. ≥ 16 points), head trauma, and the performance of definitive surgery within 6 h of ED arrival.

Secondary outcomes were evaluated as follows. Time to death during the first 28 days was estimated using the Kaplan–Meier method and compared using the log-rank test. Hazard ratios were calculated using a Cox regression model. In addition, for changes in hemoglobin levels, the *P*-value at each timepoint was calculated using a mixed model adjusted for the initial hemoglobin level, intervention, period as a fixed effect, and site and interaction of the site with the period as a random effect. A *P*-value < 0.05 was considered statistically significant. All statistical analyses were conducted using R statistical software (version 3.6.3; R Foundation for Statistical Computing).

## Results

### Study participants

**Fig. 2:**
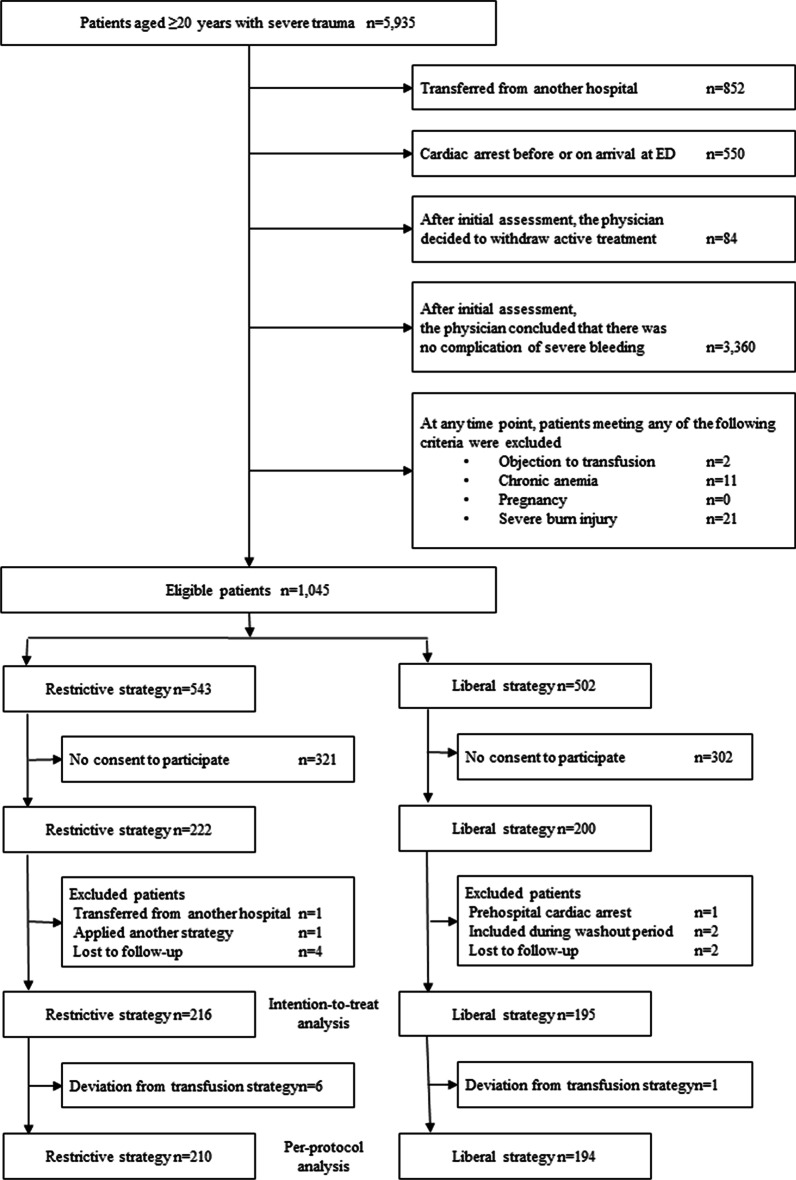
CONSORT diagram. *ED* emergency department

From May 7, 2019, to October 31, 2021, 1 045 patients were recruited in the RESTRIC trial from 22 institutions in Japan; 422 patients were enrolled. Eleven patients were excluded because of inappropriate inclusion (n = 5) or loss to follow-up (n = 6). The patients lost to follow-up were discharged alive before 28 days after admission but could not be contacted to obtain the necessary information during the observation period. The restrictive and liberal RBC strategy groups included 216 and 195 patients in the intention-to-treat analysis, respectively (Fig. 2). The number of patients included in each hospital is presented in Additional file 3. Seven patients were excluded because they deviated from the assigned transfusion strategy. Thus, 210 and 194 patients in the restrictive and liberal RBC transfusion strategy groups were included in the per-protocol analysis, respectively (Fig. 2).

Upon arrival at the ED, patient characteristics were comparable between the restrictive and liberal RBC transfusion strategy groups (Table 1). Approximately 90% of patients had blunt trauma with high Injury Severity Scores. Furthermore, major hemostatic interventions were performed in 53.7% and 66.7% of patients in the restrictive and liberal RBC transfusion strategy groups, respectively (Table 2). The frequency of major hemostatic and non-hemostatic interventions and the location within the body where the interventions were performed did not differ significantly between the groups.

**Table 1:** Patient characteristics

Characteristic	RBC transfusion strategy		P value
	Restrictive (*n* = 216)	Liberal (*n* = 195)	
Age (years), median (IQR)	61.0 (44.0–74.0)	57.0 (42.5–73.5)	0.585
Male sex, *n* (%)	145 (67.1)	130 (66.7)	1.000
Comorbidity (yes/no/unknown), *n* (%)			
Chronic heart failure	5 (2.3)/211 (97.7)/0 (0.0)	2 (1.0)/192 (98.5)/1 (0.5)	0.354
Chronic renal failure	1 (0.5)/215 (99.5)/0 (0.0)	1 (0.5)/193 (99.0)/1 (0.5)	0.737
Chronic liver failure	2 (0.9)/214 (99.1)/0 (0.0)	0 (0.0)/194 (99.5)/1 (0.5)	0.356
Chronic respiratory failure	1 (0.5)/215 (99.5)/0 (0.0)	3 (1.5)/191 (97.9)/1 (0.5)	0.231
Immunosuppression	2 (0.9)/213 (98.6)/1 (0.5)	2 (1.0)/192 (98.5)/1 (0.5)	1.000
Antithrombotic agents prior to injury, *n* (%)			
None	191 (88.4)	174 (89.2)	0.994
Antiplatelet agents	17 (7.9)	14 (7.2)	
Anticoagulation agents	3 (1.4)	3 (1.5)	
Antiplatelet + anticoagulation agents	2 (0.9)	2 (1.0)	
Unknown	3 (1.4)	2 (1.0)	
Type of injury, *n* (%)			
Blunt trauma	193 (89.4)	170 (87.2)	0.767
Penetrating trauma	22 (10.2)	24 (12.3)	
Blunt + penetrating trauma	1 (0.5)	1 (0.5)	
Time from injury to arrival at the ED (min), *n* (%)			
≤ 30	50 (23.1)	38 (19.5)	0.544
31–60	94 (43.5)	83 (42.6)	
61–90	40 (18.5)	31 (15.9)	
91–120	10 (4.6)	14 (7.2)	
> 120	8 (3.7)	11 (5.6)	
Unknown	14 (6.5)	18 (9.2)	
Injury Severity Score, median (IQR)	24.5 (14.0–34.0)	24.0 (14.0–29.0)	0.797
Abbreviated Injury Scale score, median (IQR)			
Head/neck	0.5 (0.0–3.0)	0.0 (0.0–3.0)	0.324
Face	0.0 (0.0–0.0)	0.0 (0.0–0.5)	0.356
Chest	3.0 (0.0–3.0)	2.0 (0.0–3.0)	0.368
Abdomen	2.0 (0.0–3.0)	2.0 (0.0–3.0)	0.657
Extremities/pelvic girdle	2.0 (0.0–3.0)	2.0 (0.0–3.0)	0.825
External	0.0 (0.0–1.0)	0.0 (0.0–1.0)	0.258
Physiological status on arrival at the ED, median (IQR)			
Glasgow Coma Scale score	13.0 (10.0–15.0)	13.0 (9.0–14.0)	0.159
Respiratory rate (/minute)	23.0 (19.0–28.0)	24.0 (19.0–30.0)	0.436
Heart rate (/minute)	95.0 (76.8–117.2)	94.0 (79.5–116.0)	0.916
Systolic blood pressure (mmHg)	103.5 (83.0–131.5)	110.0 (86.0–132.5)	0.557
Laboratory tests on arrival at the ED, median (IQR)			
Hemoglobin (g/dL)	12.20 (10.88–13.90)	11.90 (10.60–13.50)	0.112
Platelet count (× 10^3^/μL)	221.0 (173.8–268.2)	232.0 (179.5–278.0)	0.203
Prothrombin-INR^a^	1.080 (1.005–1.175)	1.060 (1.010–1.170)	0.678
Fibrinogen (mg/dL)^b^	215.0 (168.0–257.0)	207.0 (168.5–254.0)	0.565
Lactate (mmol/L)^c^	3.100 (2.200–4.700)	3.260 (2.085–5.208)	0.602

^a^Prothrombin-INR data were missing for one patient in the restrictive RBC transfusion strategy group

^b^Fibrinogen data were missing for one patient in the restrictive RBC transfusion strategy group

^c^Lactate data were missing for seven patients in the restrictive RBC transfusion strategy group and one patient in the liberal RBC transfusion strategy group

ED, emergency department; INR, international normalized ratio; IQR, interquartile range; RBC, red blood cell

**Table 2:** Major hemostatic and non-hemostatic interventions during the first 6 h after arrival at the ED

Intervention	RBC transfusion strategy		P value
	Restrictive (*n* = 216)	Liberal (*n* = 195)	
Major hemostatic intervention, n (%)	116 (53.7)	130 (66.7)	0.039
Site of major hemostatic intervention, n (%)			
Head	9 (4.2)	6 (3.1)	0.745
Chest	5 (2.3)	18 (9.2)	0.005
Abdomen	58 (26.9)	57 (29.2)	0.670
Pelvic fracture	29 (13.4)	36 (18.5)	0.207
Retroperitoneal hemorrhage	8 (3.7)	13 (6.7)	0.255
Extremities/neck	18 (8.3)	16 (8.2)	1.000
Other	6 (2.8)	4 (2.1)	0.754
Non-hemostatic intervention, n (%)	69 (31.9)	70 (35.9)	0.458
Site/type of non-hemostatic intervention, n (%)			
Head	13 (6.0)	15 (7.7)	0.634
Chest	1 (0.5)	0 (0.0)	1.000
Abdomen	14 (6.5)	7 (3.6)	0.269
Orthopedic surgery	44 (20.4)	51 (26.2)	0.203
Other	2 (0.9)	1 (0.5)	1.000

Both surgical and interventional radiological procedures were included

*ED* emergency department, *RBC* red blood cell

### Hemoglobin level and transfusion volume changes

**Fig. 3:**
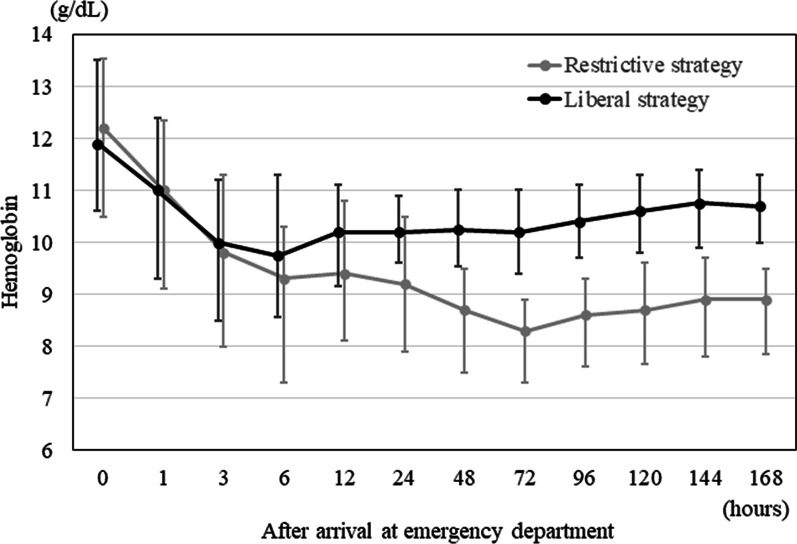
Hemoglobin levels during the first 7 days after arrival at the ED. The restrictive RBC transfusion strategy (gray) was defined as RBC transfusion with a target hemoglobin level of 7–9 g/dL. The liberal RBC transfusion strategy (black) was defined as RBC transfusion with a target hemoglobin level of 10–12 g/dL. Data are expressed as medians (interquartile ranges). *ED* emergency department, *RBC* red blood cell

In the restrictive and liberal RBC transfusion strategy groups, the hemoglobin levels upon arrival at the ED were 12.2 and 11.9 g/dL, respectively (Table 1). After arrival at the ED, hemoglobin levels rapidly decreased in both groups. The hemoglobin levels in the liberal RBC transfusion strategy group ranged from 10–11 g/dL from 3 h to 7 days after arrival at the ED. In the restrictive RBC transfusion strategy group, the hemoglobin levels from 3 h to 7 days after arrival at the ED were approximately 9 g/dL (Fig. 3).

**Fig. 4:**
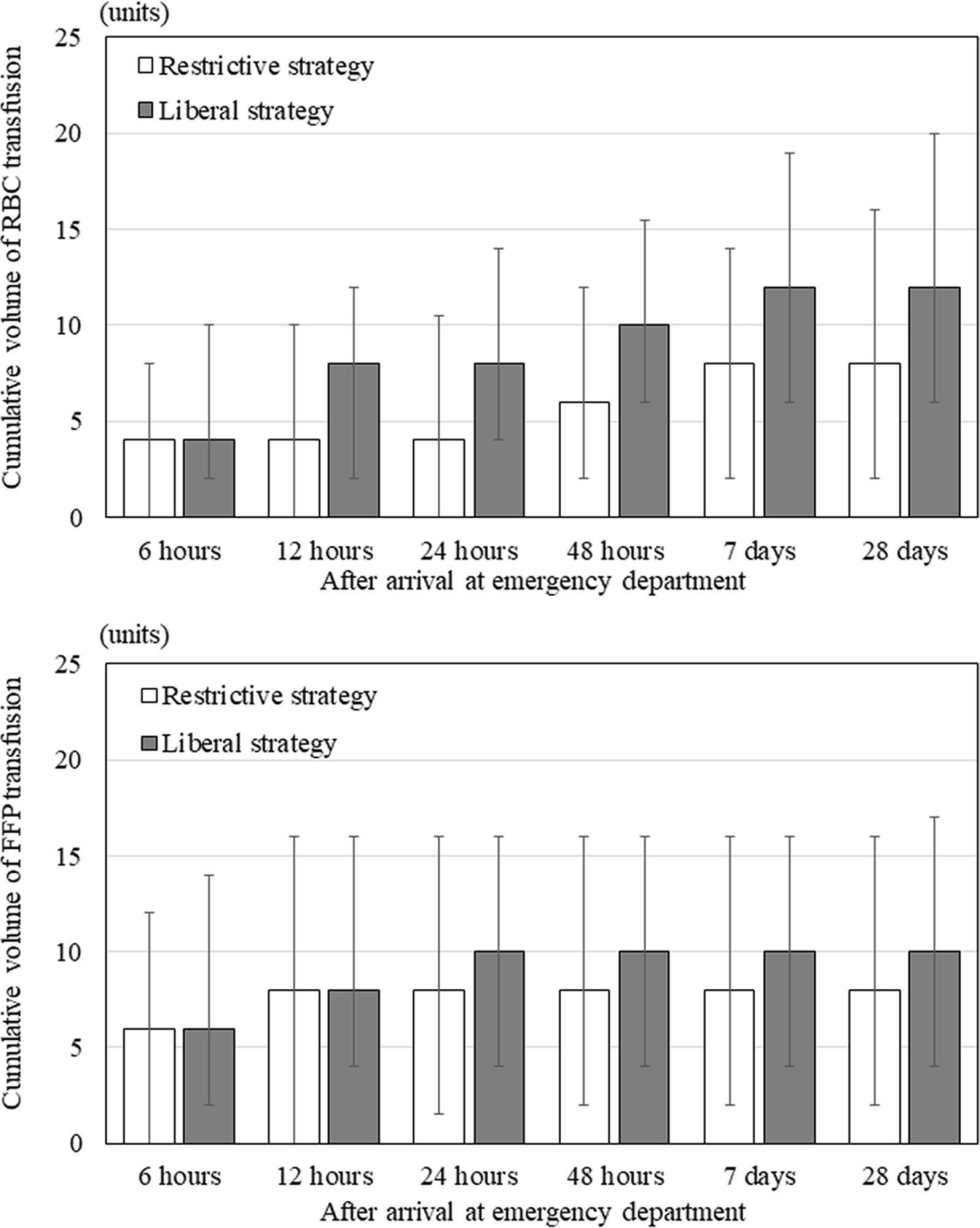
Cumulative transfusion volume during the first 28 days after arrival at the ED. White, restrictive RBC transfusion strategy; gray, liberal RBC transfusion strategy. Data are expressed as medians (interquartile ranges). *ED* emergency department, *FFP* fresh frozen plasma, *RBC* red blood cell

**Fig. 5:**
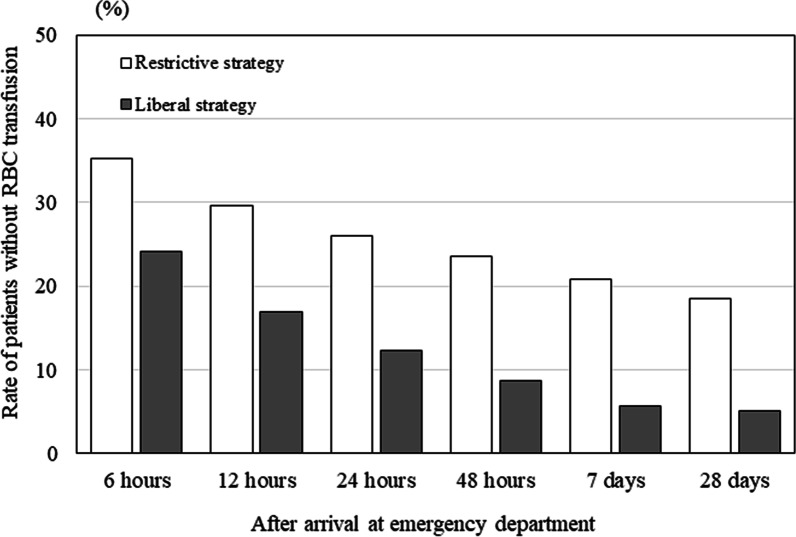
Proportion of patients without RBC transfusion. White, restrictive RBC transfusion strategy; gray, liberal RBC transfusion strategy. *RBC* red blood cell

Figure 4 shows the cumulative RBC and FFP transfusion volumes in both groups. The cumulative RBC transfusion volume during the first 6 h after arrival at the ED did not differ significantly between the groups. However, 12 h after ED arrival, the cumulative RBC transfusion volume was higher in the liberal RBC transfusion than in the restrictive RBC strategy group. The cumulative FFP transfusion volume did not differ significantly between groups during the study period. Furthermore, the cumulative platelet transfusion volumes in the restrictive and liberal RBC transfusion strategy groups were 0.0 (0.0–0.0) and 0.0 (0.0–10.0) U on Day 1, 0.0 (0.0–10.0) and 0.0 (0.0–10.0) U on Day 7, and 0.0 (0.0–10.0) and 0.0 (0.0–10.0) U on Day 28, respectively. The proportion of patients without RBC transfusion was higher in the restrictive RBC transfusion than in the liberal RBC transfusion strategy group (Fig. 5).

### Clinical outcomes

**Fig. 6:**
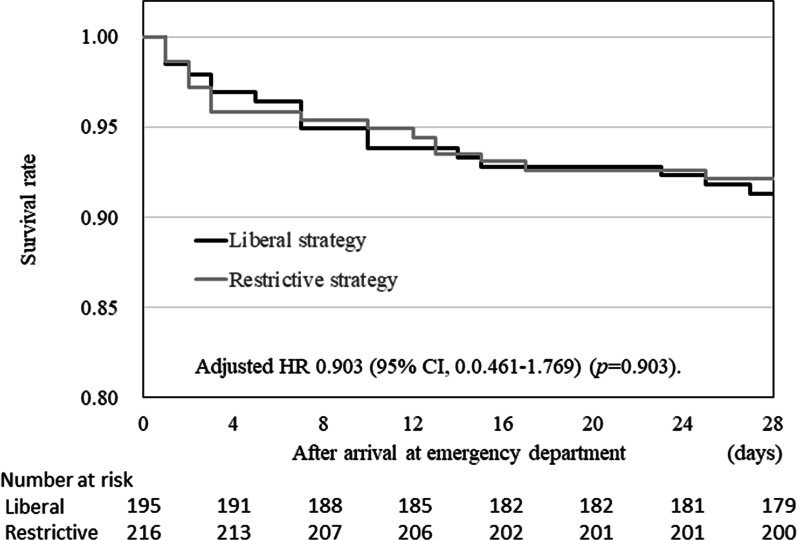
Twenty-eight-day survival Kaplan–Meier curves in the restrictive versus the liberal RBC transfusion strategy group. Gray, restrictive RBC transfusion strategy; black, liberal RBC transfusion strategy. *HR* hazard ratio, *CI* confidence interval, *RBC* red blood cell

**Fig. 7:**
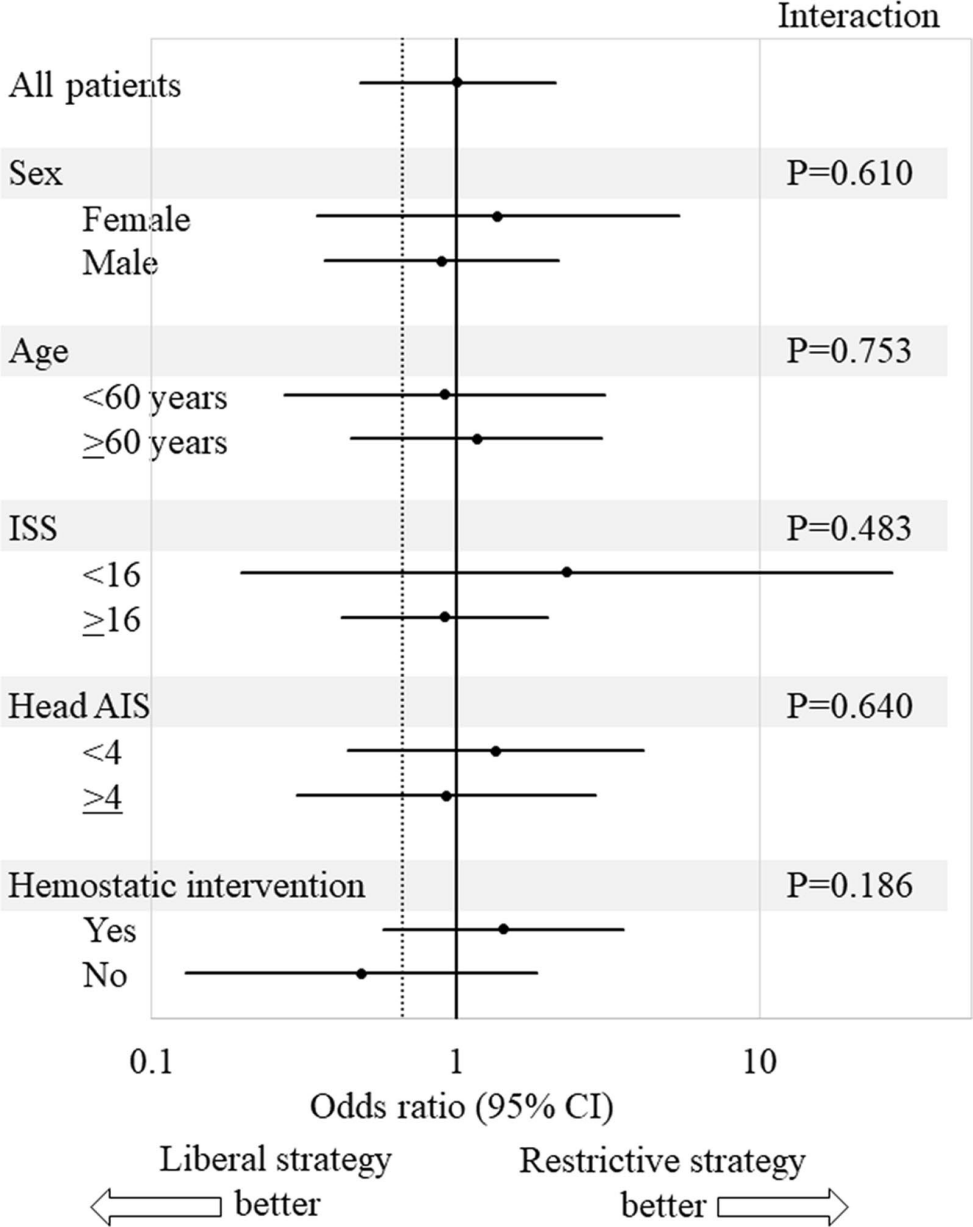
Adjusted odds ratio for the 28-day survival rate. Data are presented as unadjusted odds ratios and 95% CIs. Vertical dotted line = 0.680 (derived from a non-inferiority margin of 3% and an adjusted 28-day survival rate in the liberal RBC transfusion strategy group of 93.4%). *AIS* Abbreviated Injury Scale, *CI* confidence interval, *ISS* Injury Severity Score

The 28-day survival rates of patients who underwent restrictive and liberal RBC transfusion strategies were 92.1% and 91.3%, respectively (Table 3). The survival curves for both groups are shown in Fig. 6. The adjusted odds ratio for the 28-day survival rate of patients in the restrictive versus liberal RBC transfusion strategy group was 1.017 (95% CI: 0.485–2.131) (Fig. 7). However, the non-inferiority of the restrictive RBC transfusion strategy to the liberal RBC transfusion strategy was not confirmed because the lower 95% CI limit did not exceed 0.680 (derived from a non-inferiority margin of 3% and an adjusted 28-day survival rate in the liberal RBC transfusion strategy group of 93.4%).

**Table 3:** Survival, complications, and event-free days

Variable	RBC transfusion strategy	
	Restrictive (*n* = 216)	Liberal (*n* = 195)
28-day survival, *n* (%)	199 (92.1)	178 (91.3)
Complications during the first 7 days, *n* (%)		
Respiratory failure	23 (10.6)	18 (9.2)
Renal failure	7 (3.2)	11 (5.6)
Hepatic failure	2 (0.9)	4 (2.1)
Complications during the first 28 days, *n* (%)		
Transfusion-related acute lung injury	0 (0.0)	0 (0.0)
Cerebral infarction	5 (2.3)	2 (1.0)
Pulmonary embolism	5 (2.3)	2 (1.0)
Acute myocardial infarction	0 (0.0)	0 (0.0)
Bowel ischemia	1 (0.5)	1 (0.5)
Deep venous thrombosis	24 (11.1)	17 (8.7)
Sepsis	5 (2.3)	13 (6.7)
Event-free days during the first 28 days, median (IQR)^a^		
ICU-free days	19.0 (12.0–24.0)	19.0 (8.0–24.0)
Ventilator-free days	25.5 (18.0–28.0)	24.0 (18.0–27.0)
Catecholamine-free days	28.0 (27.0–28.0)	28.0 (27.0–28.0)

*ICU* Intensive Care Unit, *IQR* interquartile range, *RBC* red blood cell

^a^Data on event-free days were missing for one patient in the liberal RBC transfusion strategy group

No differences were noted in Glasgow Outcome Scale scores at hospital discharge between the groups (Additional file 4). The complication rates and number of event-free days did not differ significantly between the groups (Table 3). The per-protocol analysis based on patient characteristics is presented in Additional files 5, 6 and 7.

## Discussion

In this study, the application of the restrictive RBC transfusion strategy did not statistically demonstrate non-inferiority to that of the liberal RBC transfusion strategy. However, the 28-day survival rate and survival time were not significantly different between groups. Patients in the restrictive RBC transfusion strategy group had smaller RBC transfusion volumes and lower hemoglobin levels than did those in the liberal RBC transfusion strategy group.

Recently, a massive transfusion protocol comprising RBC concentrate, FFP, and platelet concentrate in a 1:1:1 ratio was recommended as aggressive coagulation support to replenish coagulation factors in patients with severe trauma [1, 2]. The massive transfusion protocols involve transfusion of the same number of units of the RBC concentrate as FFP, resulting in hemoglobin levels > 10 g/dL [1, 2]. The massive transfusion protocol is the same as the liberal RBC transfusion strategy with respect to RBC transfusion. Despite widespread acceptance of the massive transfusion strategy, the effects of the liberal RBC transfusion strategy (high hemoglobin level) have not been evaluated. The aforementioned post-hoc analysis of the TRICC trial [4] is the only study to compare the efficacies of restrictive versus liberal RBC transfusion strategies in patients with severe trauma [3]. However, the post-hoc analysis included patients admitted to the ICU and excluded those with active bleeding [3]. Therefore, the appropriate target hemoglobin level for patients with severe trauma at risk of active bleeding immediately after arrival at the ED was not investigated. This study is the first to compare the application of restrictive and liberal RBC transfusion strategies in patients with severe trauma at risk of active bleeding immediately after arrival at the ED.

In patients with severe trauma, the restrictive RBC transfusion strategy may induce ischemia and complications based on low target hemoglobin levels. The restrictive RBC transfusion strategy does not increase the incidence of ischemic complications relative to the liberal RBC transfusion strategy under the various clinical conditions or settings [4, 19–21, 24–28]. In patients with severe trauma, physicians consider the ischemic effects of low hemoglobin levels, which contribute to the risk of traumatic brain injury [29]. A randomized controlled trial [29] evaluated the effect of hemoglobin threshold levels for transfusion on neurological recovery in patients with traumatic brain injury and reported that maintaining a hemoglobin level ≥ 10 g/dL did not improve neurological outcomes. This trial excluded patients with life-threatening systemic injuries [29]. Moreover, the timing of patient inclusion was after resuscitation and not immediately after arrival at the ED. In particular, the RESTRIC trial included patients with systemic and brain injuries immediately after ED arrival; applying the restrictive RBC transfusion strategy did not have an ischemic effect on various bodily systems (including the central nervous system).

Several clinical biostatisticians were involved in the study design, and the study protocol was published [5]. Accordingly, the trial was completed as planned based on the prior sample size calculation; however, subsequently, we identified a serious miscalculation of sample size requirement. Therefore, the study was not terminated during its course. Although this study could not statistically confirm the non-inferiority of the restrictive versus liberal RBC transfusion strategy, no clinical significance between the two strategies was observed in this reasonably large cohort.

This trial has some limitations. First, the number of included patients was insufficient to confirm the non-inferiority. Second, blinding patients to the transfusion strategy was not feasible in this trial. Non-blinding may have introduced bias. Third, a cluster-randomized, crossover, non-inferiority multicenter trial cannot match the study quality of a double-blinded randomized controlled study. Fourth, the hemoglobin level for each transfusion strategy was at the target level and not at the hemoglobin threshold for initiating transfusion. Furthermore, resuscitation and transfusion were initiated in patients with major bleeding before the hemoglobin level reached the threshold. Therefore, we defined the transfusion strategy based on the target hemoglobin level and not the threshold hemoglobin level.

## Conclusions

The application of a restrictive RBC transfusion strategy for patients with severe trauma at risk of active bleeding resulted in smaller transfusion volumes and lower hemoglobin levels. Compared with those of patients in the liberal RBC transfusion strategy group, the 28-day survival rate, survival time, complication rate, and number of event-free days were not significantly different in patients in the restrictive RBC transfusion strategy group. However, the non-inferiority of the restrictive RBC transfusion strategy to the liberal RBC transfusion strategy was not statistically confirmed in terms of the 28-day survival rate.

## Supplementary Information

**Additional file 1.** Original protocol translated into English

**Additional file 2.** Participating institutions and corresponding Ethics Committees

**Additional file 3.** Number of patients included in each institution

**Additional file 4.** Glasgow Outcome Scale scores at hospital discharge

**Additional file 5.** Characteristics of patients in the per-protocol analysis

**Additional file 6.** Major hemostatic and non-hemostatic interventions during the first 6 h after arrival at the ED in the per-protocol analysis

**Additional file 7.** Survival, complications, and event-free days in the per-protocol analysis

## Data Availability

The datasets used and/or analyzed during the current study are available from the corresponding author upon reasonable request.

## Abbreviations

CI: Confidence interval
ED: Emergency department
FFP: Fresh frozen plasma
ICU: Intensive care unit
RBC: Red blood cell
RESTRIC: Restrictive Transfusion Strategy for Critically Injured Patients
TRICC: Transfusion Requirements in Critical Care
