# Supplementary material for: The Restrictive Red Blood Cell Transfusion Strategy for Critically Injured Patients (RESTRIC) trial: a cluster-randomized, crossover, non-inferiority multicenter trial of restrictive transfusion in trauma
Source: J Intensive Care. 2023 Jul 24;11:34. doi: 10.1186/s40560-023-00682-3 (PMC10364403; doi:10.1186/s40560-023-00682-3)
Supplement: Supplementary file 1 — Additional file 1. Original protocol translated into English [file 40560_2023_682_MOESM1_ESM.docx]

**Restrictive transfusion strategy for critically injured patients (RESTRIC) trial:**

**A cluster-randomized, crossover non-inferiority trial**

**Study protocol and statistical analysis plane**

**Research coordinating organization and principal investigator**

Shigeki Kushimoto

Division of Emergency and Critical Care Medicine, Tohoku University Graduate School of Medicine

**Research Office**

Mineji Hayakawa

Department of Emergency Medicine, Hokkaido University Hospital

March 28, 2018 The first edition was completed.
May 30, 2018 The version 1.1 was completed

July 11, 2018 The version 1.2 was completed
August 23rd, 2018 The version 1.3 was completed

December 3, 2018 The version 1.4 was completed

February 15, 2019 The version 1.5 was completed
May 20, 2019 The version 1.6 was completed
October 15, 2019 The version 1.7 was completed

Study period: May 7, 2019 - December 31, 2022

(Deadline for case enrollment: 2 years and 1 month after the start of case enrollment at each facility)

**Table of Contents**

[**Background** 3](#_Toc112422937)

[**Purpose of trial** 3](#_Toc112422938)

[**Summary of Test Drugs or Equipment** 4](#_Toc112422939)

[**Research Subjects and Eligibility Criteria** 4](#_Toc112422940)

[**Method of study** 5](#_Toc112422941)

[**Case Registration and Allocation Methods** 7](#_Toc112422942)

[**Observation and examination items** 7](#_Toc112422943)

[**Expected benefits and disadvantages (burdens and risks)** 11](#_Toc112422944)

[**Endpoints** 11](#_Toc112422945)

[**Discontinuation criteria and post-study actions for individual study subjects** 12](#_Toc112422946)

[**Handling of test results obtained through research on individual research subjects** 12](#_Toc112422947)

[**Handling of adverse events** 12](#_Toc112422948)

[**Approval, modification, and revision of research protocols, etc.** 13](#_Toc112422949)

[**Discontinuation, suspension, or termination of research** 14](#_Toc112422950)

[Research implementation period 14](#_Toc112422951)

[**Target Number of Patients, Basis for Setting the Target Number of Patients and Statistical Analysis Methods** 14](#_Toc112422952)

[**Consideration for human rights of research subjects** 16](#_Toc112422953)

[**Handling of personal information** 16](#_Toc112422954)

[**Method of Obtaining Consent** 16](#_Toc112422955)

[**Handling and compensation for damage to the health of research subjects** 18](#_Toc112422956)

[**Contents and Methods of Reporting to the Head of the Research Institution** 18](#_Toc112422957)

[**Effectiveness and Safety Evaluation Committee** 20](#_Toc112422958)

[**Cost Burden for Research Subjects** 20](#_Toc112422959)

[**Methods of storage and disposal of samples and information** 20](#_Toc112422960)

[**Methods of Disclosure of Research Information and Publication of Research Results** 21](#_Toc112422961)

[**Research funding and conflict of interest** 21](#_Toc112422962)

[**Monitoring** 22](#_Toc112422963)

[**Audit** 22](#_Toc112422964)

[**Research implementation system** 22](#_Toc112422965)

[**References** 25](#_Toc112422966)

# **Background**

Severe trauma patients frequently require blood transfusion due to systemic inflammatory reactions associated with trauma as well as bleeding. In a multicenter observational study led by the Japanese Association for the Surgery of Trauma, among 796 severe trauma patients with Injury Severity Score (ISS) 16 or higher, 207 (26%) patients were received red blood cell (RBC) transfusion within 6 hours, and 241 (30%) patients within 24 hours^1^. However, the optimal RBC transfusion threshold for the management of severe trauma patients during acute post-injury period is not elucidated.

The TRICC study^2^, which examined the threshold for RBC transfusion in 838 critically ill patients admitted to the ICU, included patients with hemoglobin (Hb) levels below 9.0 g/dL within 72 hours after ICU admission. The study compared the effects of Hb targets set at 10-12 g/dL and 7-9 g/dL, and observed a trend toward better survival rate and frequency of complications of organ failure in the group of patients with a target of 7-9 g/dL (restrictive transfusion group), although this was not accompanied by statistically significant differences. Furthermore, transfusion volume was significantly lower in the restrictive transfusion group. A subgroup analysis^3^ of 203 trauma patients from the TRICC study^2^ was reported. This subgroup analysis showed a decrease in RBC transfusion volume in the restrictive transfusion group, with no statistical difference in survival rate and frequency of complications of organ dysfunction. However, the TRICC study^2^ excluded patients with active bleeding, and because the study was conducted after ICU admission, transfusion strategies and hemostasis procedures (surgical hemostasis and interventional radiology (IVR)) prior to ICU admission were not considered. Therefore, it may be inappropriate to apply the results of the TRICC study as a basis for transfusion strategies in the acute post-injury period.

Patients with traumatic brain injury (TBI) are independent targets for restrictive transfusion therapy in terms of the possibility of improving prognosis by increasing the oxygen supply to the brain. The Epo Severe TBI Trial was an RCT that examined the effects of restrictive transfusion strategy in patients with closed traumatic brain injury^4^. The neurological outcome in the restricted transfusion group strategy tended to be better than that of the liberal transfusion strategy group, although the difference was not statistically significant. Similar RCTs examining transfusion thresholds for TBI are also registered on Clinical trials.gov (NCT02203292, NCT02968654, NCT03260478).

# **Purpose of trial**

To examine the impact of a restrictive transfusion strategy versus a liberal transfusion strategy on survival outcome, transfusion volume, and complication of organ dysfunction in severe trauma patients.

# **Summary of Test Drugs or Equipment**

Not applicable

# **Research Subjects and Eligibility Criteria**

Subjects (1) are eligible if they meet all of the selection criteria (2) and none of the exclusion criteria (3).

(1) Subjects.

Trauma patients transferred to emergency departments of the hospital participating in this trial

(2) Selection criteria

1. Persons who are 20 years of age or older at the time of obtaining consent.
2.
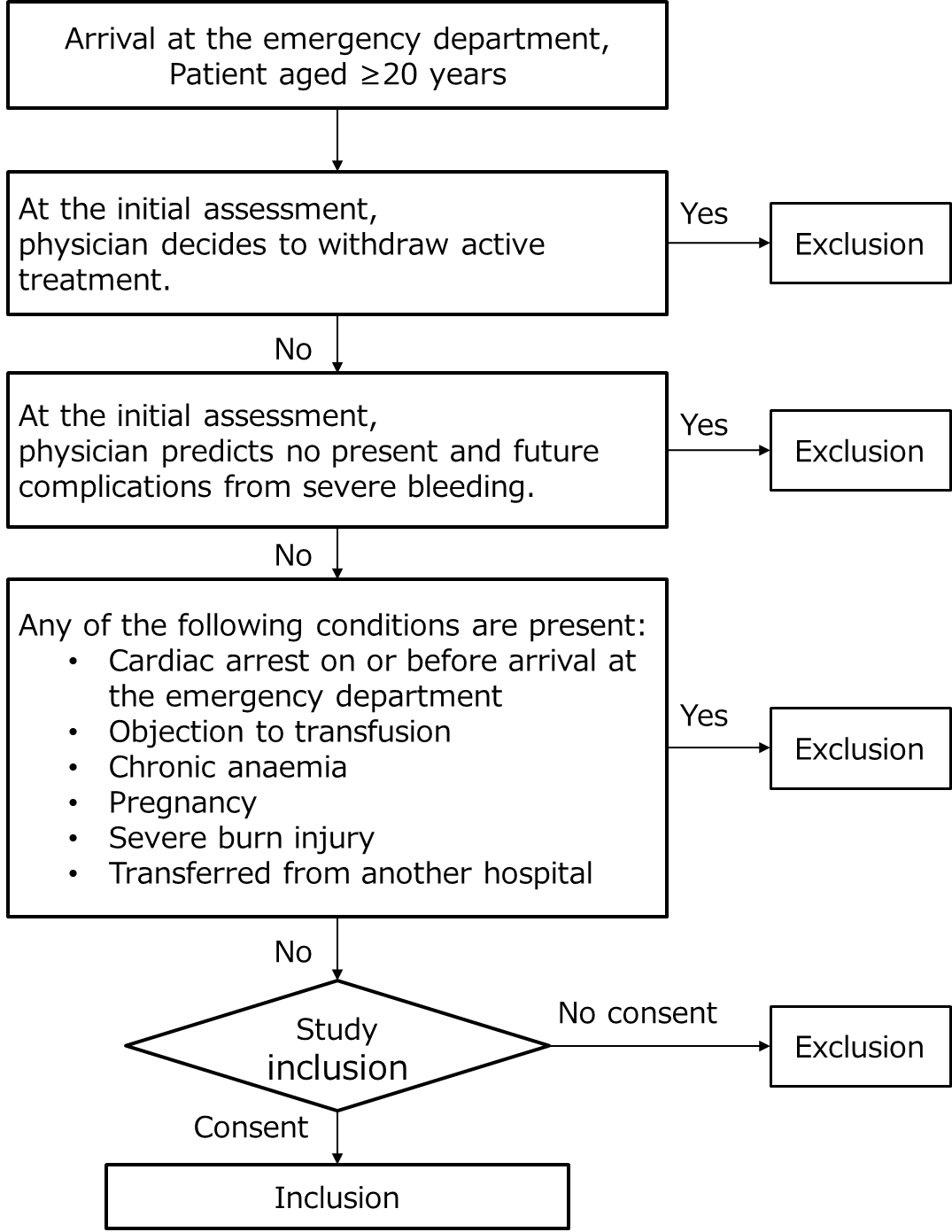
Patients who have been or will be judged by the physician in charge to have bleeding that could cause unstable circulation, or patients who have been or will be judged by the physician in charge to have a risk of such bleeding due to acute surgical procedures.
3. Patients who have been fully informed about the participation in this study, and who have given their or their next of kin's free and voluntary written consent based on full understanding.

(3) Exclusion Criteria

1. Cardiac arrest before or upon arrival at the hospital,
2. Transfer from another hospital,
3. Physician’s decision to withdraw active treatment at the initial assessment,
4. Severe burn injuries (≥15% of the body surface),
5. Pregnancy,
6. Chronic anaemia (Hb level ≤7 g/dL) and
7. Known objection to blood transfusions.
8. Other patients deemed inappropriate as research subjects by the investigator

The above selection/exclusion criteria will also take into account the time progression after admission to emergency department. Refer to the flowchart for the relationship between the time lapse and the selection and exclusion criteria.

(4) Research subjects for whom consent by a surrogate is required and the reasons for such consent

This study will include research subjects who may have difficulty obtaining valid informed consent. This is because we believe that this research is concerned with events that are unique to the diseases that are the primary focus of this study. The following persons will be selected as surrogates, based on their ability to represent the wishes and interests of the research subjects, taking into consideration the family structure, etc. of the research subjects.

The research subject's spouse, adult children, adult siblings or grandchildren, grandparents, relatives living in the same household, or persons considered to be equivalent to such close relatives. Of these, the closest possible relative shall be selected and requested as a surrogate.

# **Method of study**

(1) Study type and design

Cluster randomized crossover non-inferiority trial

(2) Outline of the study

This study is a cluster-randomized trial in which facilities will be assigned to two transfusion strategies: a liberal transfusion strategy and a restrictive transfusion strategy. After the participating are determined, the facilities will be randomized and assigned to either the liberal transfusion strategy or the restrictive transfusion strategy.

The target Hb level in the liberal transfusion strategy is 10-12 g/dL, while the target Hb level for the restrictive transfusion strategy is 7-9 g/dL. This target Hb level is only a goal, not a criterion
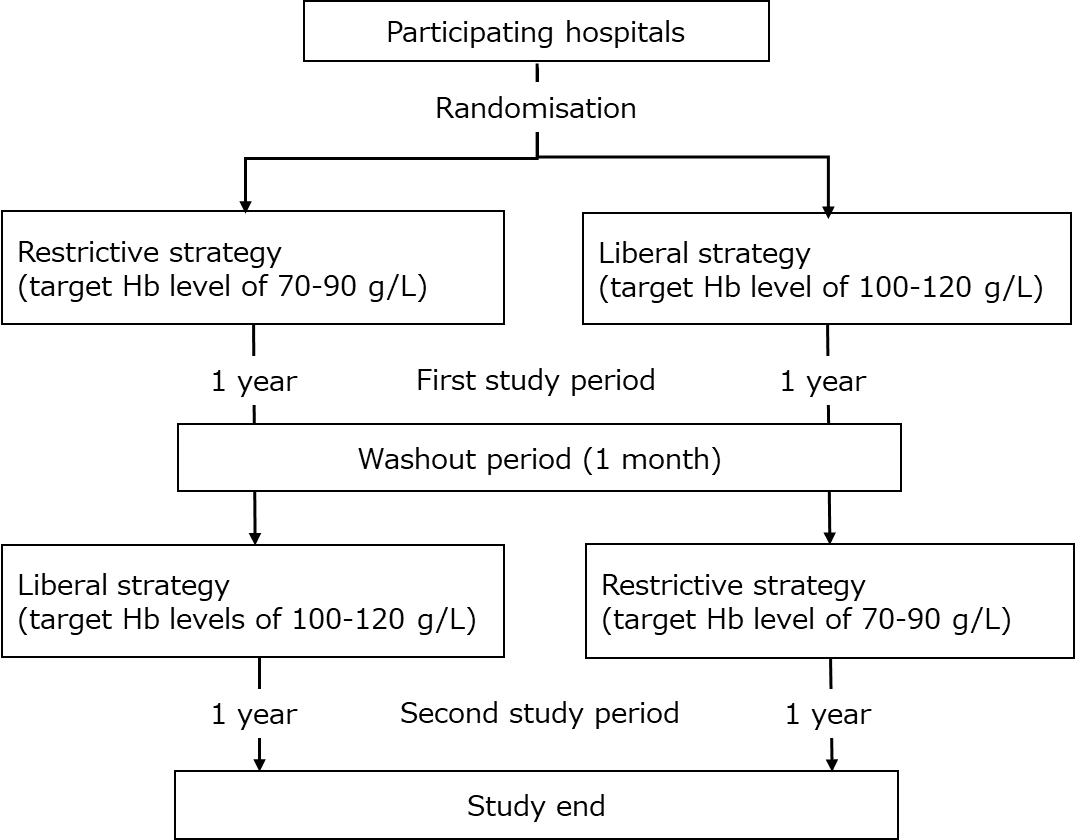
for initiating transfusion, and the timing of transfusion initiation, speed of transfusion, and other factors should be determined by clinical judgment. The timing and speed of transfusion should be determined based on clinical judgment. Either of the RBC transfusion strategies will be applied to patients until (1) 7 days after admission to the hospital, (2) discharge from the ICU, (3) decision to withdraw active treatment, or (4) death.

In this study, various treatments, including hemostasis, will be performed as needed, at the discretion of the physician in charge.

After the randomization of facilities, the facilities will apply the first transfusion strategy for 1 year (first study period). After a washout period of 1 month after the end of the first study period, the second transfusion strategy will be applied for another 1 year (second study period;).

(3) Eligibility Criteria for Research Participation

Facilities eligible to participate in this study shall be those that meet all of the following criteria

1. The facility treats patients with severe traumatic injuries.
2. Approval of this research plan has been obtained from the independent clinical research review committee of the institution or the appropriate review committee of each medical institution (hereinafter referred to as the "review committee").
3. Consent for participation in this study has been submitted to the research office by the head of the facility or the head of the department.

(4) Method of administration of test drug or use of test equipment

Not applicable

(5) Regulations on concomitant medications (therapies)

Not applicable

(6) Provisions for dose reduction and withdrawal

Not applicable

(7) Expected period of study participation by study subjects

The observation period of 28 days after the injury will be the study participation period.

# **Case Registration and Allocation Methods**

1. Site enrollment/allocation method

After confirming the eligibility criteria of the participating sites, the allocation staff at the Research office will allocate the transfusion strategies to each site. The allocation of transfusion strategies to each site will be done in a research office, according to a pre-designed random assignment list, in the order of their decision to participate in the study. The allocation list will be kept by the staff in charge of allocation and will not be disclosed to the principal investigator or research assistants.

The principal investigator at each site will receive confirmation of eligibility for participation in the study from the research office and will receive an enrollment confirmation form that includes the transfusion strategy to be selected and its duration.

In addition, the principal investigator at each site will disclose information regarding each site's participation in the study, the target hemoglobin levels and their duration in the manner prescribed by each site.

1. Case allocation method

This study is a cluster-randomized trial with clusters of participating facility. The transfusion strategy for each facility will be determined prior to the admission of patients to each facility. Therefore, no case-by-case assignment will be made. When patients are admitted into the emergency department in each facility, consent for the study will be obtained as soon as possible, and the transfusion strategy assigned to each facility will be applied.

1. Blinding
   There will be no blinding.

# **Observation and examination items**

1. Facility Information

Nature of hospital (university hospital or non-university hospital)
Number of beds in the hospital
Nature of ICU

Number of ICU beds where severe trauma patients are initially admitted

Availability of emergency transfusion of O(+) red blood cells

Availability of AB-FFP emergency transfusion

Availability of cryoprecipitate

Availability of fibrinogen concentrate

Availability of high volume transfusion protocols

Average number of emergency physicians per trauma initial care

Number of trauma patients with ISS ≥9 in 2017

Availability of bedside testing (hemoglobin, fibrinogen, other coagulation system tests)

1. Characteristics of patient

Age, gender, date of injury, time from injury to arrival at emergency department

Trauma classification (blunt/penetrating), presence/absence of antithrombotic medication, presence/absence of comorbidities, and mechanisms of injury

1. Pathophysiologic information on arrival at emergency department

Systolic blood pressure, respiratory rate, heart rate, Glasgow Coma Scale, RTS (calculated value)

1. Examination and treatment

FAST, surgery/IVR for hemostasis within 6 hours, other surgery within 6 hours

1. TBSS-related information (missing measurements allowed)
   Number of FAST positive sites, systolic blood pressure after 1L infusion, pelvic fracture (AO classification)
2. Injury and severity
   Head and neck AIS, head-only AIS (excluding pure DAI), facial AIS, thoracic AIS, abdominal AIS, Pelvic AIS, body surface AIS (excluding hypothermia), ISS (calculated value), TRISS (calculated value)
3. Laboratory tests on arrival at emergency department

Time of blood collection, platelet count, hemoglobin level, PT-INR, fibrinogen, lactate level

1. Hemoglobin level

First hemoglobin level measured at the following time points
The first blood collection ~1hr, 1~3, 3~6, 6~12, 12~24, 24~48, 48~72, 72~96, 96~120, 120~144, 144~168hr after the arrival at emergency department

(The measurement method can be either hemocytometer or blood gas analyzer.)

1. Blood transfusion volume

Total amount of red blood cells, fresh frozen plasma, platelets, cryoprecipitate, and fibrinogen concentrate at 6 hours, 12 hours, 24 hours, 48 hours, 7 days, and 28 days

1. Complications of organ failure within 7 days of admission (worst case after ICU admission)

Renal failure (KDIGO classification Stage 3)
Respiratory failure (PEEP ≥ 5 and P/F ≤ 200 according to the Berlin definition)
Liver failure (SOFA ≥ 3 points, T.Bil ≥ 6mg/dL)

1. Information during 28 days after the admission

Date of ICU discharge, date of hospital discharge

Survival after 28 days

Event-free days (ventilator, catecholamine, ICU stay)

Complications during hospitalization (up to 28 days)

DVT, PE, stroke, myocardial infarction, intestinal ischemia, Sepsis (yes/no)

TRALI (TRALI/possible/no complications)

1. Information at discharge

Date of hospital discharge, place of transfer after hospital discharge (including discharge by death), number of days in hospital (calculated value)
Glasgow Outcome Scale (GOS) at discharge

1. Intentional deviation from the hemoglobin target

Intentional deviation from the hemoglobin target, if any, timing and details

1. Withdrawal of active treatment

If the patient chose to withdraw from active treatment during the observation period, the date and time of the decision of withdrawal.

Date and time of hospital admission.

The above data will be collected using Electronic Data Capture (NorthNet).

Schedule of evaluation

|  | Observation period （28days of hospital discharge） | | | | | | | |
| --- | --- | --- | --- | --- | --- | --- | --- | --- |
| Timing | On arrival | 6hr | 12hr | 24hr | 48hr | 7日 | Hospital discharge | 28日 |
| Consent | ○ |  |  |  |  |  |  |  |
| Characteristics of patients | ○ |  |  |  |  |  |  |  |
| Pathophysiologic information | ○ |  |  |  |  |  |  |  |
| Examination and treatment | ←○→ | |  |  |  |  |  |  |
| Injury and severity | ○ |  |  |  |  |  |  |  |
| TBSS-related information | ○ |  |  |  |  |  |  |  |
| Laboratory tests | ○ |  |  |  |  |  |  |  |
| Hemoglobin level | ←○→ | | | | | |  |  |
| Blood transfusion volume |  | ○ | ○ | ○ | ○ | ○ |  | ○ |
| Complications of organ failure | ←○→ | | | | | |  |  |
| Survival after 28 days |  |  |  |  |  |  |  | ○ |
| Event-free days | ←○→ | | | | | | |  |
| Complications | ←○→ | | | | | | |  |
| Date of hospital discharge |  |  |  |  |  |  | ○ |  |
| place of transfer after hospital discharge |  |  |  |  |  |  | ○ |  |
| GOS |  |  |  |  |  |  | ○ |  |
| Intentional deviation from the hemoglobin target | ←○→ | | | | | |  |  |
| Withdrawal of active treatment | ←○→ | | | | | |  |  |

# **Expected benefits and disadvantages (burdens and risks)**

1. Expected benefits

In the case of restrictive transfusion strategy, the frequency of transfusion may decrease and transfusion-related complications may decrease. On the other hand, in the case of the liberal transfusion strategy, oxygen carrying capacity may be increased and organ damage may be avoided. There are no obvious burdens associated with conducting the study.

1. Expected disadvantages (burden and risk)

In the case of the restrictive transfusion strategy, there is a risk of decreased oxygen carrying capacity and organ damage due to low hemoglobin levels. On the other hand, in the case of the liberal transfusion strategy, there is a risk of increased transfusion-related complications due to increased transfusion frequency. There are no obvious burdens associated with conducting the study.

1. Comprehensive evaluation of benefits and disadvantages, and countermeasures against disadvantages

The balance of expected disadvantages in both groups is within an acceptable range based on current medical knowledge.

# **Endpoints**

1. Primary endpoint

Survival rate at 28 days after hospital admission

1. Secondary endpoints
2. Survival time during 28 days after admission to the hospital
3. In-hospital survival rate
4. Total transfusion volume of red blood cells, fresh frozen plasma, and platelets (1, 7, and 28 days after admission to the hospital)
5. Event-free days (ventilator, catecholamine, ICU stay) in 28 days after admission to the hospital
6. Complications of organ failure
7. Complications during hospitalization (up to 28 days after admission to the hospital
8. GOS at discharge

# **Discontinuation criteria and post-study actions for individual study subjects**

1. Actions at the discontinuation of the study

If the principal investigator determines that it is not feasible to continue the research on an individual research subject for any of the following reasons, the research on that research subject will be discontinued. In such cases, the reason for discontinuation will be explained to the research subjects as necessary. The treatment of the research subject after discontinuation will be handled in good faith so as not to disadvantage the research subject.

1. Criteria for discontinuation
2. If the research subject requests to withdraw from participation in the research or withdraws his/her consent.
3. When this entire study is terminated.
4. When the investigator deems it appropriate to discontinue the research for other reasons.
5. Post-study actions

After the research, the principal investigator will provide medical care deemed most appropriate for the research subjects, including the results are obtained in this research.

# **Handling of test results obtained through research on individual research subjects**

Results of various tests will be disclosed to patients in the same manner as in routine medical care.

# **Handling of adverse events**

1. Treatment of research subjects in the event of an adverse event

An adverse event is defined as any unwanted or unintended injury or illness or its symptoms (including abnormal values of laboratory tests) that occurs in a research subject, regardless of whether or not there is a causal relationship to the research being conducted.

When an adverse event is recognized, the investigator shall immediately take appropriate treatments and document the adverse event in the medical record. If treatment for the adverse event becomes necessary, the investigator will inform the research subject.

1. Reporting of serious adverse events

Serious adverse event is defined as follows. However, those that are clearly directly attributable to trauma are excluded.

1. Causing death
2. Life-threatening
3. Requiring hospitalization or prolonged hospitalization for treatment
4. That which results in permanent or marked disability or dysfunction
5. Inherited birth defects in offspring

When the investigator recognizes the occurrence of a serious adverse event, the investigator will take necessary measures, such as explaining the event to the research subjects, and will promptly report the event to the principal investigator.

When the principal Investigator receives the report of the occurrence of a serious adverse event, the principal Investigator shall promptly report it to the director of the research institution, take appropriate action, and promptly share the information regarding the occurrence of the serious adverse event with other investigators.

Possible adverse events include cerebral infarction, liver injury, acute lung injury, acute kidney injury, gastrointestinal injury, heart failure, arrhythmia, sepsis, transfusion-related complications (hemolytic side effects, transfusion-related acute lung injury, transfusion-related circulatory overload, anaphylaxis, post-transfusion GVHD, hyperkalemia).

1. Reported Significant Adverse Events

Not applicable

1. Other adverse events

Other adverse events will be documented by the investigator in the medical record, etc., as appropriate.

**Approval, modification, and revision of research protocols, etc.**

The principal investigator shall submit the research protocol, etc. to the head of the research institution in advance, and obtain the approval of the review committee and the permission of the head of the research institution with regard to the implementation of the research. In case of modification or revision of the research protocol, etc., the principal investigator shall promptly submit the revised version to the head of the research institution in accordance with the established procedures, and obtain the approval of the review committee and the permission of the head of the research institution.

# **Discontinuation, suspension, or termination of research**

1. Discontinuation or suspension of research

The investigator shall consider whether or not to continue the implementation of the research if any of the following applies.

1. When matters concerning safety and efficacy or other important information become known.
2. When it is judged to be extremely difficult to reach the planned number of cases due to difficulties in recruiting research subjects.
3. When the purpose of the research has been achieved before the planned number of cases or planned period is reached.
4. When the Review Committee has instructed the investigator to change the research plan, etc., and the investigator finds it difficult to accept the change.

The principal investigator will discontinue the research if the review committee recommends or directs discontinuation of the research. When the decision to discontinue or suspend the research is made, the decision shall be promptly reported in writing to the head of the research institution, together with the reason for the discontinuance or suspension.

1. Termination of research

Upon completion of the research, the principal investigator shall promptly submit a report on the completion of the research to the head of the research institution.

# Research implementation period

From May 7, 2019 to December 31, 2022 (Deadline for case registration: 2 years and 1 month after the start of case registration at each institution)

# **Target Number of Patients, Basis for Setting the Target Number of Patients and Statistical Analysis Methods**

1. Target number of patients, number of participating sites, and rationale for setting the target number of patients

The total number of patients in the entire study is 400, consisting of 200 patients with the restrictive transfusion strategy and 200 patients with the liberal transfusion strategy. A total of cases at our hospital is 30.

Rationale

In a multicenter observational study (15 centers, 1 year data) led by t the Japanese Association for the Surgery of Trauma, 241 of 796 patients with severe trauma received RBC transfusion within 24 hours, and the 28-day mortality rate was approximately 25%^1^. Assuming this mortality rate to be that of the liberal transfusion strategy group, and setting the non-inferiority margin to 3%, ICCc and ICCp to both 0.05, and 17 participating centers to demonstrate non-inferiority of the restrictive transfusion strategy in the primary endpoint of 28-day survival, the number of patients required to achieve a power of 80% at a one-sided significance level of 2.5% would be 170 in each group. The target number of patients was set at 200 for each group, for a total of 400 cases, to allow for the possibility of dropouts and ineligible cases.

The calculation method for the number of cases was based on a previous report.^5^ Based on past data, this number of patients can be included in two years.

1. Statistical analysis method

A mixed-effects model will be used for the primary outcome, with random effects for facility and facility x timing and fixed effects for timing and transfusion strategy.^6^ P0 and P1 will be the 28-day survival rates of the control and intervention groups, respectively, and the null hypothesis H0: P0 - P1 < 0.03 will be tested and confidence intervals calculated to determine non-inferiority will be determined. The primary analysis will be based on the largest analysis population (Full Analysis Set), and patients with missing 28-day survival, the primary endpoint, will be treated as dropouts and excluded from the analysis of the primary endpoint. The primary analysis will include cases with intentional deviations of hemoglobin target (intention-to-treat analysis), while a per protocol analysis will be performed excluding cases with intentional deviations of hemoglobin target as a secondary analysis.

For the secondary endpoint (1), a survival curve will be generated by the Kaplan-Meier method and examined by the log-rank test. (2) The number of in-hospital survivors will be counted. (3) Summary statistics of total transfusion volume of RBC, fresh-frozen plasma (1, 7, and 28 days) at each time point will be calculated and plotted on a graph over time. (4) Summary statistics for 28 event-free days (ventilator, catecholamine, ICU stay) will be calculated. (5) Tally the presence or absence of complications of each organ failure. (6) Tally complications. (7) Tally GOS at discharge. In each analysis, missing measures will be excluded.

Subgroup analyses will be performed to identify differences in the effect of transfusion strategies on pre-specified subgroups (gender: male and female, age: <60 or ≥60 years, ISS: <16 or ≥16, with or without head trauma [head AIS ≥4] complications, with or without hemostatic surgery/IVR within 6 hours). Estimates of treatment effects and p-values will be calculated for each subgroup. We will perform analyses for both models with all subgroups as covariates (adjusted) and models that do not include other covariates (unadjusted).

If the planned number of sites and subjects differs from the actual number of sites and subjects, a power analysis may be conducted post hoc.

# **Consideration for human rights of research subjects**

All personnel in charge of this research will conduct the research in compliance with the "Declaration of Helsinki (amended October 2013)" and the "Ethical Guidelines for Medical Research Involving Human Subjects (Ministry of Education, Culture, Sports, Science and Technology, Ministry of Health, Labour and Welfare Notification No. 3, 2014).

# **Handling of personal information**

Information related to the implementation of research will be managed by replacing names, addresses, etc. with completely different control numbers (research IDs) so that it is not possible to determine who the information belongs to at a glance.

The principal investigator will supervise the strict safekeeping of the correspondence list. This control number should be used when the data is provided to joint research institutions and collaborators.

When the principal investigators release information obtained through this research, they should not include information that could identify research subjects.

# **Method of Obtaining Consent**

The investigators will give the research subject (including a surrogate if one is needed; the same shall apply hereinafter) the consent document approved by the Review Committee, provide sufficient explanation in writing and orally, and obtain consent in writing of the subject's free will. The investigators shall exercise caution in obtaining consent. If consent is obtained from a surrogate, the investigator will explain the study to the patient himself/herself again when the patient's general condition has improved and the patient has sufficient capacity to make decisions, and will endeavor to obtain consent.

When information that may affect the consent of research subjects is obtained, or when changes are made to the research plan, etc. that may affect the consent of research subjects, the investigators will promptly provide information to the research subjects, re-confirm in advance their willingness to participate in the research, and obtain prior approval from the review committee.

1. The name of the research and the fact that permission to conduct the research has been obtained from the head of the research institution.
2. The name of the research institution and the name of the principal investigator (when research is conducted jointly with other research institutions, the name of the joint research institution and the name of the principal investigator of the joint research institution are included)
3. Purpose and significance of the research
4. Methods of the research (including the purpose of use of the samples/information obtained from the research subjects) and duration
5. Reasons for selection as research subjects
6. Burdens to be incurred by the research subjects and anticipated risks and benefits
7. A statement that consent to the implementation or continuation of the research may be withdrawn at any time (if there are cases in which it will be difficult to take measures in accordance with the content of the withdrawal by the research subject, etc., a statement to that effect and the reasons therefor)
8. That the research subjects, etc. will not be treated disadvantageously by refusing to consent to the implementation or continuation of the research or by withdrawing their consent.
9. Method of disclosure of information concerning the research
10. A statement that research protocols and materials on research methods may be obtained or viewed upon request by research subjects, etc., to the extent that this does not hinder the protection of the personal information of other research subjects, etc., or ensure the originality of the research, and the method of obtaining or viewing such materials.
11. Handling of personal information, etc. (including the method of anonymization, if any, and the creation of anonymized or de-identified processed information, if any)
12. Methods of storage and disposal of samples and information
13. Sources of funding for research, etc., conflicts of interest related to research at the research institution and personal earnings, etc., and the status of conflicts of interest related to research by researchers, etc.
14. Responses to consultations, etc., from research subjects, etc., and other persons concerned
15. In the case of research involving invasive procedures, whether or not compensation will be provided for any damage to health caused by the research, and the details of such compensation
16. If there is a possibility that the sample/information obtained from the research subject will be used for future research that is not specified at the time consent is obtained from the research subject, etc., or will be provided to other research institutions, a statement to that effect and the details of such possible use or provision at the time consent is obtained.
17. A statement that, on the assumption that the confidentiality of the research subject will be maintained, the persons engaged in monitoring and the review committee will have access to the samples and information concerning the research subject to the extent necessary.

The following items are not applicable to this research and are therefore omitted.

1. If there is any financial burden or gratuity to the research subjects, a statement to that effect and the details thereof
2. If the research involves medical treatment that exceeds normal medical care, matters concerning other treatment methods, etc.
3. In the case of research involving medical treatment that exceeds normal medical care, measures to be taken regarding the provision of medical care to the research subjects after the research is conducted.
4. If the implementation of the research may lead to important findings concerning the health of the research subjects, genetic characteristics that may be passed on to their offspring, etc., the results of the research (including incidental findings) pertaining to the research subjects.

# **Handling and compensation for damage to the health of research subjects**

If a health hazard occurs to a research subject as a result of the implementation of this research, the investigators will take appropriate measures. In such cases, if treatment or tests are necessary, they will be performed within the research subject's normal insurance coverage. In light of the above, this research will not provide financial compensation for any health damage to the research subjects. This point shall be explained to the research subjects and their understanding shall be obtained.

# **Contents and Methods of Reporting to the Head of the Research Institution**

1. Reports on progress, etc.

At least once a year, the principal investigator shall report in writing to the head of the research institution on the progress of the research and the occurrence of adverse events associated with the implementation of the research.

1. Report of serious adverse events

If the principal investigator recognize the occurrence of a serious adverse event in the conduct of research involving invasive procedures, he/she will promptly report it to the head of the research institution.

1. When information on facts, etc. that undermine the ethical validity or scientific rationality of the research is obtained

If the principal investigator obtains facts or information that undermine the ethical validity or scientific rationality of the research, or information that is likely to undermine such facts or information and is considered to affect the continuation of the research, the principal investigator shall report to that effect to the head of the research institution without delay.

1. When information is obtained that undermines the appropriateness of the conduct of the research or the reliability of the research results

If the investigator obtains facts or information that undermine or may undermine the appropriateness of the implementation of the research or the reliability of the research results, the person in charge of the research shall promptly report to that effect to the head of the research institution.

1. Report of termination of research (including the case of discontinuation; the same shall apply hereinafter)

When the research is terminated, the principal investigator shall report to that effect and a summary of the results of the research to the head of the research institution without delay in writing in accordance with “Discontinuation, suspension, or termination of research”.

1. Status of management of samples and information used in research

The principal investigator shall manage the storage of samples and information obtained from human subjects as necessary in accordance with “Methods of storage and disposal of samples and information”, and report to the head of the research institution on the status of management.

1. Reporting of publication of research results

When the final publication of the results is made, the principal investigator reports to the head of the research institution without delay in accordance with “Methods of Disclosure of Research Information and Publication of Research Results”. If, after reporting that the final publication has been made, it is decided to publish the results of the research, the principal investigator will promptly report this to the head of the research institution.

# **Effectiveness and Safety Evaluation Committee**

The principal investigator will report his/her views and responses to the reported adverse event to the Effectiveness and Safety Evaluation Committee, which is a third party, and request a review of the appropriateness of the principal investigator's views and responses. The Efficacy and Safety Evaluation Committee will review and consider the contents of the report and make a recommendation in writing to the principal investigator regarding future actions.

# **Cost Burden for Research Subjects**

Since the administration of pharmaceuticals used in this research and the tests performed will be covered by insurance, there will be no cost burden on research subjects as a result of their participation in the research.

# **Methods of storage and disposal of samples and information**

1. Records of transfer of samples and information

When transferring samples and information to/from collaborating research institutions and persons who only provide existing samples and information, a record of the transfer of samples and information (name of the research institution to which the samples and information are provided, name of the principal investigator at the research institution to which the samples and information are provided, name of the source institution, name of the principal investigator at the source institution, items of samples and information, history of acquisition of the samples and information, etc.) must be kept in the records of the research plan and the research plan.

1. Methods of storage and disposal of samples

Not applicable.

1. Methods of storage and disposal of information, etc.

The principal investigator will be instructed to ensure the accuracy of the information (including records of transfer of samples and information to and from other research institutions), and to manage the information as necessary to prevent leakage, mixing, theft, loss, etc. of the information, etc.

Information, etc. obtained in this research shall be stored in a lockable cabinet in the emergency department office.

The principal investigator will keep the information, etc. used in the study for as long as possible, and will store it appropriately until at least five years have passed since the date on which the completion of the study was reported, or until three years have passed since the date on which the final publication of the results of the study was reported, whichever is later.

When disposing of the samples and information, they shall be anonymized and handled with care in terms of personal information.

1. Secondary use of samples and information

Information on research subjects obtained in this study may be used for future research that is not specified at the time consent is obtained. In such cases, a new research protocol will be prepared or modified, and approval from the review committee and permission from the head of the research institution will be obtained as necessary.

When information is to be provided to other research institutions, it should be reported to the head of the research institution and anonymized before being provided.

# **Methods of Disclosure of Research Information and Publication of Research Results**

The principal investigator shall register an outline of the relevant research in the public database prior to its implementation, and shall update it as appropriate according to changes in the research protocol and the progress of the research. Upon completion of the research, the results of such research will be registered without delay. When the results are made public, necessary measures shall be taken to protect the human rights of the research subjects, etc. and other persons concerned, or the rights and interests of the research subjects, etc. and other persons concerned. When the final publication of the results is made, it shall be reported to the head of the research institution without delay.

The public database to be registered shall be the University Hospital Medical Information Network (UMIN-CTR).

# **Research funding and conflict of interest**

This research will be conducted with research funds from the department to which the principal investigator belongs. The handling of conflict of interest screening will be conducted in accordance with the regulations of each institution. The person in charge of the research at Hokkaido University Hospital shall report the necessary matters to the Conflict of Interest Review Committee for its review and approval in accordance with the provisions of the "Internal Rules on Conflict of Interest Management for Clinical Research at Hokkaido University Hospital.

If intellectual property rights arise from this research, such rights shall belong to the researcher and not to the research subjects.

# **Monitoring**

The principal investigator shall conduct monitoring to ensure the credibility of the research and to confirm that the human rights, safety, and welfare of research subjects are protected, that this research is being conducted in compliance with the research protocol, and that the data reported by research personnel are accurately collected. The person in charge of monitoring shall conduct monitoring in accordance with the monitoring procedures prepared in advance.

# **Audit**

　　　　　Not applicable.

# **Research implementation system**

This research will be conducted under the following structure.

**Principal investigator (who is ultimately responsible for the research)**

Professor Shigeki Kushimoto, MD, PhD

Division of Emergency and Critical Care Medicine, Tohoku University Graduate School of Medicine

Aoba-ku, Seiryo-cho, 1-1, Sendai city 980-8574, Japan

TEL: 022-717-7489 FAX: 022-717-7492

E-mail: [kussie@emergency-medicine.med.tohoku.ac.jp](mailto:kussie@emergency-medicine.med.tohoku.ac.jp)

**Research Office**

Lecturer Mineji Hayakawa, MD, PhD

Department of Emergency Medicine, Hokkaido University Hospital

Kita-ku, N14 W5, Sapporo 060-8648, Japan

TEL: 011-706-7377 FAX: 011-706-7378

E-mail: [mineji@dream.com](mailto:mineji@dream.com)

**Research Office, Allocation Manager**
Takashi Tagami, MD, MPH, PhD

Department of Emergency and Critical Care Medicine, Nippon Medical School Musashi Kosugi Hospital

Department of Clinical Epidemiology and Health Economics, School of Public Health, The University of Tokyo

Nakahara-ku, Kosugi-cho, 1-396, Kawasaki city, Kanagawa 211-8533, Japan

TEL: 044-733-5181

E-mail: [t-tagami@nms.ac.jp](mailto:t-tagami@nms.ac.jp)

**Data Management Facilities**

Department of Emergency Medicine, Hokkaido University Hospital

Kita-ku, N14 W5, Sapporo 060-8648, Japan

TEL: 011-706-7377 FAX: 011-706-7378

**Statistical analysis supervisor**
Takashi Tagami, MD, MPH, PhD

Department of Emergency and Critical Care Medicine, Nippon Medical School Musashi Kosugi Hospital

Department of Clinical Epidemiology and Health Economics, School of Public Health, The University of Tokyo

Nakahara-ku, Kosugi-cho, 1-396, Kawasaki city, Kanagawa 211-8533, Japan

TEL: +81-44-733-5181

E-mail: [t-tagami@nms.ac.jp](mailto:t-tagami@nms.ac.jp)

**Statistical Analysis Advisor**

Hiroaki Iijima, MPH

Astellas Amgen Biopharma Inc.

Sapia Tower, 1-7-12 Marunouchi, Chiyoda-ku, Tokyo 100-0005, Japan

TEL: 03-5293-9866 FAX: 03-6730-9645

E-mail: hiroaki.iijima@mail.utoronto.ca
(Transferred from Division of Biostatistics, Clinical Research Development Center, Hokkaido University Hospital in October 2018)
Hiroaki Iijima does not handle personal information.

**Monitoring Facilities**
Department of Emergency Medicine, Hokkaido University Hospital

Kita-ku, N14 W5, Sapporo 060-8648, Japan

TEL: 011-706-7377 FAX: 011-706-7378

**Efficacy and Safety Evaluation Committee**

Dr. Akio Kimura, MD, PhD

President of the Japanese Association for the Surgery of Trauma

Director, Emergency Medical Center

National Center for Global Health and Medicine Emergency Center

1-21-1 Toyama, Shinjuku-ku, Tokyo 162-8655, Japan

TEL: 03-3202-7181

E-mail: [akimura@hosp.ncgm.go.jp](mailto:akimura@hosp.ncgm.go.jp)

Professor Daizo Saito, MD, MPH, PhD

Department of Trauma Research, National Defense Medical Center, National Defense Medical College

3-2 Namiki, Tokorozawa-shi, Saitama 359-8513, Japan

TEL: 04-2995-1211

E-mail: [ds0711@ndmc.ac.jp](mailto:ds0711@ndmc.ac.jp)

**Participating Institutions**

| Department of Emergency Medicine, Hokkaido University Hospital | Mineji Hayakawa, MD, PhD |
| --- | --- |
| Department of Emergency and Critical Care Medicine, Tohoku University Hospital | Shigeki Kushimoto, MD, PhD |
| Advanced Critical Care and Emergency Centre, Okayama University Hospital | Tetsuya Yumoto, MD, PhD |
| Department of Emergency and Critical Care Medicine, Juntendo University Urayasu Hospital | Yutaka Kondo, MD, PhD |
| Department of Emergency and Critical Care Medicine, Chiba University Graduate School of Medicine | Takeo Kurita, MD |
| Trauma and Acute Critical Care Centre, Tokyo Medical and Dental University Hospital of Medicine | Keita Nakatsutsumi, MD, PhD |
| Department of Emergency and Critical Care Medicine, Japan Red Cross Maebashi Hospital | Kenji Fuzitsuka, MD |
| Department of Emergency and Critical Care Medicine, Tokyo Saiseikai Central Hospital | Shiho Irino, MD, MPH |
| Department of Emergency Medicine, Division of Acute Care Surgery, Teikyo University School of Medicine | Kaori Ito, MD, PhD |
| Department of Emergency and Critical Care Medicine, Nippon Medical School Tama Nagayama Hospital | Fumihiko Nakayama, MD |
| Department of Emergency and Critical Care Medicine, Fukuoka University Hospital | Yuhei Irie, MD, PhD |
| Shock and Trauma Centre, Nippon Medical School Chiba Hokusoh Hospital | Taichiro Ueda, MD |
| Department of Emergency and Critical Care Medicine, Wakayama Medical University | Yuko Okishio, MD |
| Advanced Critical Care Centre, Gifu University Hospital | Sho Nachi, MD |
| Department of Acute Care Surgery, Shimane University Faculty of Medicine | Tomohiro Muronoi, MD, PhD |
| Department of Emergency Medicine, Gunma University Graduate School of Medicine | Makoto Aoki, MD, PhD |
| Advanced Trauma, Emergency and Critical Care Centre, Oita University Hospital | Tomotaka Shibata, MD, PhD |
| Senri Critical Care Medical Centre, Saiseikai Senri Hospital | Yusuke Ito, MD |
| Senshu Trauma and Critical Care Centre, Rinku General Medical Centre | Hiroshi Fukuma, MD |
| Emergency and Critical Care Centre, Kochi Health Sciences Centre | Yuichi Saisaka, MD |
| Advanced Emergency and Critical Care Centre, Saitama Red Cross Hospital | Katsura Hayakawa, MD |
| Department of Emergency and Critical Care Medicine, Nippon Medical School | Naoki Tominaga, MD |

**Implementation System at Tohoku University Hospital**

| Principal Investigator |  |
| --- | --- |
| Division of Emergency and Critical Care Medicine, Tohoku University Graduate School of Medicine | Professor Shigeki Kushimoto |
| Research Members |  |
| Division of Emergency and Critical Care Medicine, Tohoku University Graduate School of Medicine | Lecturer Daisuke Kudo |
| Division of Emergency and Critical Care Medicine, Tohoku University Graduate School of Medicine | Assistant Professor Yu kawazoe |
| Department of Emergency and Critical Care Medicine, Tohoku University Hospital | Lecturer Takashi Irinoda |
| Department of Emergency and Critical Care Medicine, Tohoku University Hospital | Assistant Professor Motoki Fujita |
| Department of Emergency and Critical Care Medicine, Tohoku University Hospital | Assistant Professor Yoriko Miyagawa |

How to enroll study subjects (participants) and what to do in case of adverse events

Daisuke Kudo, MD, PhD

Division of Emergency and Critical Care Medicine, Tohoku University Graduate School of Medicine

Phone: 022-717-7489 E-mail: [kudodaisuke@med.tohoku.ac.jp](mailto:kudodaisuke@med.tohoku.ac.jp)

# **References**

1. Maekawa K. Determination of optimal cutoff values of hemoglobin, platelet count and fibrinogen at 24 hours after injury associated with mortality in trauma patients. *Journal of Japanese Association for The Surgery of Trauma (in Japanese)*. 2016;30(3):412-418.

2. Hebert PC, Wells G, Blajchman MA, et al. A multicenter, randomized, controlled clinical trial of transfusion requirements in critical care. Transfusion Requirements in Critical Care Investigators, Canadian Critical Care Trials Group. *N Engl J Med*. 1999;340(6):409-417.

3. McIntyre L, Hebert PC, Wells G, et al. Is a restrictive transfusion strategy safe for resuscitated and critically ill trauma patients? *J Trauma*. 2004;57(3):563-568; discussion 568.

4. Robertson CS, Hannay HJ, Yamal JM, et al. Effect of erythropoietin and transfusion threshold on neurological recovery after traumatic brain injury: a randomized clinical trial. *JAMA*. 2014;312(1):36-47.

5. Giraudeau B, Ravaud P, Donner A. Sample size calculation for cluster randomized cross-over trials. *Stat Med*. 2008;27(27):5578-5585.

6. Morgan KE, Forbes AB, Keogh RH, Jairath V, Kahan BC. Choosing appropriate analysis methods for cluster randomised cross-over trials with a binary outcome. *Stat Med*. 2017;36(2):318-333.
