# Supplementary material for: The Restrictive Red Blood Cell Transfusion Strategy for Critically Injured Patients (RESTRIC) trial: a cluster-randomized, crossover, non-inferiority multicenter trial of restrictive transfusion in trauma
Source: J Intensive Care. 2023 Jul 24;11:34. doi: 10.1186/s40560-023-00682-3 (PMC10364403; doi:10.1186/s40560-023-00682-3)
Supplement: Supplementary file 2 — Additional file 2. Participating institutions and corresponding Ethics Committees [file 40560_2023_682_MOESM2_ESM.docx]

**Additional file 2.** Participating institutions and corresponding ethics committees

| **Participating institution** | **Ethics committee** |
| --- | --- |
| **Principal institution** | |
| Department of Emergency and Critical Care Medicine, Tohoku University Hospital | Ethics Committee of Tohoku University Graduate School of Medicine |
| **Project management institution** | |
| Department of Emergency Medicine, Hokkaido University Hospital | The Institutional Review Board of Hokkaido University Hospital |
| **Other participating institutions** | |
| Advanced Critical Care and Emergency Center, Okayama University Hospital | Okayama University Graduate School of Medicine, Dentistry and Pharmaceutical Sciences and Okayama University Hospital, Ethics Committee |
| Advanced Critical Care Center, Gifu University Hospital | Medical Review Board of Gifu University Graduate School of Medicine |
| Advanced Emergency and Critical Care Center, Saitama Red Cross Hospital | Hospital Ethical Committee of Saitama Red Cross |
| Advanced Trauma, Emergency and Critical Care Center, Oita University Hospital | The Institutional Review Board of Interventional Clinical Research of Oita University Hospital |
| Department of Emergency Medicine, Gunma University Graduate School of Medicine | Institutional Review Board of Gunma University Hospital |
| Department of Acute Care Surgery, Shimane University Faculty of Medicine | The Shimane University Institutional Committee on Ethics |
| Department of Emergency and Critical Care Medicine, Chiba University Graduate School of Medicine | Chiba University Certified Clinical Research Review Board |
| Department of Emergency and Critical Care Medicine, Fukuoka University Hospital | Institutional Review Board of Fukuoka University Hospital |
| Department of Emergency and Critical Care Medicine, Japan Red Cross Maebashi Hospital | Research Review Board of Japan Red Cross Maebashi Hospital |
| Department of Emergency and Critical Care Medicine, Juntendo University Urayasu Hospital | The Ethics Committee of the Juntendo University Urayasu Hospital |
| Department of Emergency and Critical Care Medicine, Nippon Medical School | Ethics Committee of Nippon Medical School Hospital |
| Department of Emergency and Critical Care Medicine, Nippon Medical School Tama Nagayama Hospital | Ethics Committee of Nippon Medical School Tamanagayama Hospital |
| Department of Emergency and Critical Care Medicine, Tokyo Saiseikai Central Hospital | Research Ethics Committee, Tokyo Saiseikai Central Hospital |
| Department of Emergency and Critical Care Medicine, Wakayama Medical University | The Ethics Review Board of Wakayama Medical University |
| Department of Emergency Medicine, Division of Acute Care Surgery, Teikyo University School of Medicine | Teikyo University Institutional Review Board |
| Emergency and Critical Care Center, Kochi Health Sciences Center | Institutional Review Board, Kochi Health Sciences Center |
| Senri Critical Care Medical Center, Saiseikai Senri Hospital | Ethics Committee of Saiseikai Senri Hospital |
| Senshu Trauma and Critical Care Center, Rinku General Medical Center | Ethics Committee for Clinical Research, Rinku General Medical Center |
| Shock and Trauma Center, Nippon Medical School Chiba Hokusoh Hospital | The Ethical Review Board of Nippon Medical School Chiba Hokusoh Hospital |
| Trauma and Acute Critical Care Center, Tokyo Medical and Dental University Hospital of Medicine | Medical Research Institute Tokyo Medical and Dental University |
