# Supplementary material for: The Restrictive Red Blood Cell Transfusion Strategy for Critically Injured Patients (RESTRIC) trial: a cluster-randomized, crossover, non-inferiority multicenter trial of restrictive transfusion in trauma
Source: J Intensive Care. 2023 Jul 24;11:34. doi: 10.1186/s40560-023-00682-3 (PMC10364403; doi:10.1186/s40560-023-00682-3)
Supplement: Supplementary file 3 — Additional file 3. Number of patients included in each institution [file 40560_2023_682_MOESM3_ESM.docx]

**Additional file 3.** Number of patients included in each institution

| **Institution No.** | **Red blood cell transfusion strategy** | |
| --- | --- | --- |
|  | **Restrictive (*n=*216)**  n (%) | **Liberal (*n=*195)**  n (%) |
| 1 | 28 (57.1) | 21 (42.9) |
| 2 | 22 (64.7) | 12 (35.3) |
| 3 | 20 (54.1) | 17 (45.9) |
| 4 | 14 (42.4) | 19 (57.6) |
| 5 | 10 (32.3) | 21 (67.7) |
| 6 | 17 (60.7) | 11 (39.3) |
| 7 | 16 (57.1) | 12 (42.9) |
| 8 | 12 (44.4) | 15 (55.6) |
| 9 | 13 (50) | 13 (50) |
| 10 | 7 (31.8) | 15 (68.2) |
| 11 | 8 (42.1) | 11 (57.9) |
| 12 | 11 (61.1) | 7 (38.9) |
| 13 | 6 (54.5) | 5 (45.5) |
| 14 | 8 (88.9) | 1 (11.1) |
| 15 | 5 (62.5) | 3 (37.5) |
| 16 | 6 (75) | 2 (25) |
| 17 | 8 (100) | 0 (0) |
| 18 | 3 (42.9) | 4 (57.1) |
| 19 | 0 (0) | 3 (100) |
| 20 | 2 (100) | 0 (0) |
| 21 | 0 (0) | 2 (100) |
| 22 | 0 (0) | 1 (100) |
