# Supplementary figures and images for: The Restrictive Red Blood Cell Transfusion Strategy for Critically Injured Patients (RESTRIC) trial: a cluster-randomized, crossover, non-inferiority multicenter trial of restrictive transfusion in trauma
Source: J Intensive Care. 2023 Jul 24;11:34. doi: 10.1186/s40560-023-00682-3 (PMC10364403; doi:10.1186/s40560-023-00682-3)

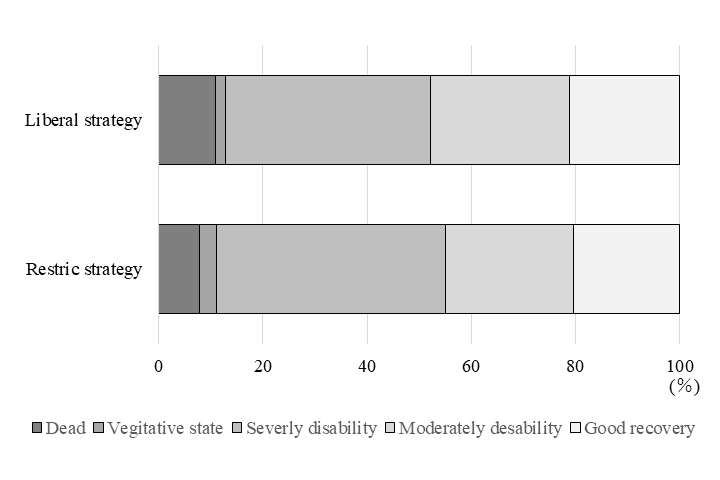

Supplement: Supplementary file 4 — Additional file 4. Glasgow Outcome Scale scores at hospital discharge [file 40560_2023_682_MOESM4_ESM.tif]
