# Supplementary material for: The Restrictive Red Blood Cell Transfusion Strategy for Critically Injured Patients (RESTRIC) trial: a cluster-randomized, crossover, non-inferiority multicenter trial of restrictive transfusion in trauma
Source: J Intensive Care. 2023 Jul 24;11:34. doi: 10.1186/s40560-023-00682-3 (PMC10364403; doi:10.1186/s40560-023-00682-3)
Supplement: Supplementary file 5 — Additional file 5. Characteristics of patients in the per-protocol analysis [file 40560_2023_682_MOESM5_ESM.docx]

**Additional file 5.** Characteristics of patients in the per-protocol analysis

| **Characteristic** | **RBC transfusion strategy** | |
| --- | --- | --- |
|  | **Restrictive (*n*=210)** | **Liberal (*n*=194)** |
| Age (years), median (IQR) | 61.0 (44.2–74.0) | 57.0 (42.2–73.0) |
| Male sex, n (%) | 142 (67.6) | 129 (66.5) |
| Comorbidity (yes/no/unknown), n (%) | | |
| Chronic heart failure | 5 (2.4) | 2 (1.0) |
| Chronic renal failure | 1 (0.5) | 1 (0.5) |
| Chronic liver failure | 2 (1.0) | 0 (0.0) |
| Chronic respiratory failure | 1 (0.5) | 3 (1.5) |
| Immunosuppression | 2 (1.0) | 2 (1.0) |
| Antithrombotic agents prior to injury, n (%) | | |
| None | 185 (88.1) | 173 (89.2) |
| Antiplatelet agents | 17 (8.1) | 14 (7.2) |
| Anticoagulation agents | 3 (1.4) | 3 (1.5) |
| Both | 2 (1.0) | 2 (1.0) |
| Unknown | 3 (1.4) | 2 (1.0) |
| Type of injury, n (%) |  |  |
| Blunt trauma | 188 (89.5) | 169 (87.1) |
| Penetrating trauma | 21(10.0) | 24 (12.4) |
| Blunt and penetrating trauma | 1 (0.5) | 1 (0.5) |
| Time from injury to arrival at the ED (min), n (%) | | |
| <30 | 50 (23.8) | 37 (19.1) |
| 31–60 | 91 (43.3) | 83 (42.8) |
| 61–90 | 38 (18.1) | 31 (16.0) |
| 91–120 | 10 (4.8) | 14 (7.2) |
| >120 | 8 (3.8) | 11 (5.7) |
| Unknown | 13 (6.2) | 18 (9.3) |
| Injury Severity Score, median (IQR) | 24.0 (14.0–34.0) | 24.5 (14.5–29.0) |
| Abbreviated Injury Scale score, median (IQR) | | |
| Head/neck | 0.0 (0.0–3.0) | 0.0 (0.0–3.0) |
| Face | 0.0 (0.0–0.0) | 0.0 (0.0–0.8) |
| Chest | 3.0 (0.0–3.0) | 2.0 (0.0–3.0) |
| Abdomen | 2.0 (0.0–3.0) | 2.0 (0.0–3.0) |
| Extremities/pelvic girdle | 2.0 (0.0–3.0) | 2.0 (0.0–3.0) |
| External | 0.0 (0.0–1.0) | 0.0 (0.0–1.0) |
| Physiological status on arrival at the ED | | |
| Glasgow Coma Scale score, median (IQR) | 13.0 (10.0–15.0) | 13.0 (9.0–14.0) |
| Respiratory rate (/min), median (IQR) | 23.0 (19.0–28.0) | 24.0 (19.0–30.0) |
| Heart rate (/min), median (IQR) | 95.0 (77.0–117.8) | 94.0 (79.2–116.0) |
| Systolic blood pressure (mmHg), median (IQR) | 103.0 (83.0–130.0) | 110.0 (86.0–132.0) |
| Laboratory tests on arrival at the ED | | |
| Hemoglobin (g/dL), median (IQR) | 12.20 (10.83–13.90) | 11.95 (10.62–13.50) |
| Platelet count (×10^3^ /μL), median (IQR) | 219.5 (172.2–262.2) | 232.0 (180.0–279.0) |
| Prothrombin-INR, median (IQR)^a^ | 1.080 (1.010–1.180) | 1.065 (1.010–1.170) |
| Fibrinogen (mg/dL), median (IQR)^b^ | 214.0 (168.0–257.0) | 207.0 (167.8–253.0) |
| Lactate (mmol/L), median (IQR)^c^ | 3.100 (2.205–4.855) | 3.300 (2.100–5.217) |

ED, emergency department; INR, international normalized ratio; IQR, interquartile range; RBC, red blood cell.

^a^ Prothrombin-INR data were missing for one patient in the restrictive strategy group.

^b^ Fibrinogen data were missing for one patient in the restrictive strategy group.

^c^ Lactate data were missing for seven patients in the restrictive strategy group and one patient in the liberal strategy group.
