# Supplementary material for: The Restrictive Red Blood Cell Transfusion Strategy for Critically Injured Patients (RESTRIC) trial: a cluster-randomized, crossover, non-inferiority multicenter trial of restrictive transfusion in trauma
Source: J Intensive Care. 2023 Jul 24;11:34. doi: 10.1186/s40560-023-00682-3 (PMC10364403; doi:10.1186/s40560-023-00682-3)
Supplement: Supplementary file 6 — Additional file 6. Major hemostatic and non-hemostatic interventions during the first 6 h after arrival at the ED in the per-protocol analysis [file 40560_2023_682_MOESM6_ESM.docx]

**Additional file 6.** Major hemostatic and non-hemostatic interventions during the first 6 h after arrival at the ED in the per-protocol analysis

| **Intervention** | **RBC transfusion strategy** | |
| --- | --- | --- |
|  | **Restrictive (*n*=210)** | **Liberal (*n*=194)** |
| Major hemostatic interventions, n (%) | 110 (52.4) | 129 (66.5) |
| Site of major hemostatic intervention, n (%) | | |
| Head | 8 (3.8) | 6 (3.1) |
| Chest | 5 (2.4) | 18 (9.3) |
| Abdomen | 55 (26.2) | 56 (28.9) |
| Pelvic fracture | 28 (13.3) | 36 (18.6) |
| Retroperitoneal hemorrhage | 7 (3.3) | 13 (6.7) |
| Extremities/neck | 18 (8.6) | 16 (8.2) |
| Other |  |  |
| Non-hemostatic intervention, n (%) | 66 (31.4) | 70 (36.1) |
| Site/type of non-hemostatic intervention, n (%) | | |
| Head | 12 (5.7) | 15 (7.7) |
| Chest | 1 (0.5) | 0 (0.0) |
| Abdomen | 13 (6.2) | 7 (3.6) |
| Orthopedic surgery | 43 (20.5) | 51 (26.3) |
| Other | 2 (1.0) | 1 (0.5) |

ED, emergency department; RBC, red blood cell.

Both surgical and interventional radiological procedures were included.
