# Supplementary material for: The Restrictive Red Blood Cell Transfusion Strategy for Critically Injured Patients (RESTRIC) trial: a cluster-randomized, crossover, non-inferiority multicenter trial of restrictive transfusion in trauma
Source: J Intensive Care. 2023 Jul 24;11:34. doi: 10.1186/s40560-023-00682-3 (PMC10364403; doi:10.1186/s40560-023-00682-3)
Supplement: Supplementary file 7 — Additional file 7. Survival, complications, and event-free days in the per-protocol analysis [file 40560_2023_682_MOESM7_ESM.docx]

**Additional file 7.** Survival, complications, and event-free days in the per-protocol analysis

| **Variable** | **RBC transfusion strategy** | |
| --- | --- | --- |
|  | **Restrictive (*n*=210)** | **Liberal (*n*=194)** |
| 28-day survival, n (%) | 193 (91.9) | 177 (91.2) |
| Complications during the first 28 days, n (%) | | |
| Transfusion-related acute lung injury | 0 (0.0) | 0 (0.0) |
| Cerebral infarction | 5 (2.4) | 2 (1.0) |
| Pulmonary embolism | 5 (2.4) | 2 (1.0) |
| Acute myocardial infarction | 0 (0.0) | 0 (0.0) |
| Bowel ischemia | 1 (0.5) | 1 (0.5) |
| Deep venous thrombosis | 24 (11.4) | 17 (8.8) |
| Sepsis | 5 (2.4) | 13 (6.7) |
| Event-free days during the first 28 days, median (IQR)^a^ | | |
| ICU-free days | 19.0 (12.0–24.0) | 19.0 (8.0–24.0) |
| Ventilator-free days | 26.0 (18.0–28.0) | 24.0 (18.0–27.0) |
| Catecholamine-free days | 28.0 (27.0–28.0) | 28.0 (27.0–28.0) |

ICU, intensive care unit; IQR, interquartile range; RBC, red blood cell.

^a^ Data on event-free days were missing for one patient in the liberal strategy group.
